# Biomarker-Based Pharmacological Characterization of ENX-102, a Novel α2/3/5 Subtype-Selective GABAA Receptor Positive Allo-Steric Modulator: Translational Insights from Rodent and Human Studies

**DOI:** 10.3390/cells14201575

**Published:** 2025-10-10

**Authors:** Pauline Nettesheim, Krishna C. Vadodaria, Kimberly E. Vanover, Laura G. J. M. Borghans, Estibaliz Arce, William Brubaker, Stephen Cunningham, Stephanie Parks, Jordi Serrats, Vikram Sudarsan, Eve Taylor, Erica Klaassen, Frederik E. Stuurman, Gabriel E. Jacobs

**Affiliations:** 1 Centre for Human Drug Research, 2333 CL Leiden, The Netherlands; p.nettesheim@lumc.nl (P.N.); lborghans@chdr.nl (L.G.J.M.B.);; 2 Department of Clinical Pharmacy and Toxicology, Leiden University Medical Center, 2333 ZG Leiden, The Netherlands; 3 Engrail Therapeutics, Inc., San Diego, CA 92130, USA; krishna@engrail.com (K.C.V.); bill.brubaker@engrail.com (W.B.); lurgan@optonline.net (S.C.); stephanie.parks@engrail.com (S.P.); jordiserrats@gmail.com (J.S.); vikram@engrail.com (V.S.); eve.taylor@engrail.com (E.T.); 4 Department of Psychiatry, Leiden University Medical Center, 2333 ZG Leiden, The Netherlands

**Keywords:** GABA_A_ receptor, benzodiazepine, subtype-selective, ENX-102, translational

## Abstract

Gamma-aminobutyric acid type A receptors (GABA_A_Rs) are pentameric ligand-gated ion channels essential for inhibitory neurotransmission in the central nervous system. Subtype-specific expression patterns of GABA_A_R subunits underlie their diverse roles in regulating anxiety, motor function, and sedation. While non-selective GABA_A_R positive allosteric modulators (PAMs), such as benzodiazepines, are clinically effective anxiolytic drugs, their non-selective activity across α1/2/3/5 subunit-containing GABA_A_Rs leads to sedation, cognitive impairment, and risk of dependence. To address this, we evaluated ENX-102, a novel GABA_A_R PAM, which exhibits selectivity for α2/3/5 subunits. In rodents, ENX-102 demonstrated dose-dependent anxiolytic-like activity following acute and sub-chronic administration, without sedation. ENX-102 exhibited a dose-dependent quantitative electroencephalography (qEEG) spectral signature in rodents that was distinct from that of benzodiazepines. In a double-blind, placebo-controlled, multiple-ascending dose study in healthy human volunteers, ENX-102 was evaluated using the NeuroCart, a CNS test battery including saccadic peak velocity (SPV), adaptive tracking, pupillometry, body sway, the Bond and Lader Visual Analog Scale (VAS), the Visual Verbal Learning Task (VVLT), and qEEG. ENX-102 produced reductions in SPV that were indicative of central target engagement, with minimal effects on alertness and motor coordination, which is consistent with subtype-selective GABA_A_R targeting. Notably, qEEG revealed increased β-band power and decreased δ- and θ-band activity, which were distinct from the spectral profile of non-selective PAMs, supporting translational alignment with preclinical findings. Across dose levels, ENX-102 was well tolerated and exhibited favorable pharmacokinetics. These results support further clinical development of ENX-102 as a next-generation GABA_A_R subtype-selective anxiolytic drug.

## 1. Introduction

Gamma-aminobutyric acid (GABA) represents the primary inhibitory neurotransmitter in the human central nervous system (CNS). It is essential for maintaining neurophysiological and behavioral homeostasis in typically dynamic and potentially emotionally challenging environments, and acts as an endogenous ligand at GABA_A_, GABA_B_, and GABA_C_ receptors. GABA_A_ receptors (GABA_A_Rs) are expressed across diverse neural circuits, where they crucially regulate phasic GABA-mediated CNS inhibition [1]. As a consequence, dysregulation of GABAergic signaling through GABA_A_Rs has been implicated in hyperaroused states and/or cortical hyperexcitability, including anxiety, trauma-related disorders, and epilepsy [2,3].

GABA_A_Rs are ion-gated channels composed of five transmembrane subunits expressed in different configurations in diverse neurocircuits. Although 19 distinct GABA_A_R subunits (α1–6, β1–3, γ1–3, δ, ε, θ, π, and ρ1–3) have been identified [4], its predominant isoform consists of a single γ, two α, and two β subunits. Such structural and anatomical heterogeneity allows for diverse GABA-mediated physiological effects, making GABA_A_Rs essential in various CNS functions, depending on the subtype combination and receptor expression site. In both rodents and humans, α1 subunit-containing receptors display a wide expression pattern with relatively greater density in the cerebral cortex, where they contribute to non-specific CNS suppression, which leads to reduced alertness or sedation [5,6,7]. In contrast, α2 and possibly α3 subunits display a different expression pattern with relatively greater expression in subcortical limbic structures such as the amygdala, potentially contributing to attenuation of hyperarousal and emotional response. Although α5 subunit-containing GABA_A_Rs expression is generally lower in the CNS, with a less restricted expression in the hippocampus in rodents, they have been implicated in memory function [8,9,10]. Due to the widespread CNS distribution of GABA_A_Rs, non-selective positive allosteric modulators (PAMs) such as benzodiazepines have shown broad efficacy in diverse neuropsychiatric disorders, particularly as anxiolytics [11]. However, their non-selective targeting of α1/2/3/5 subunit-containing GABA_A_Rs is associated with unwanted side-effects including sedation, cognitive impairment, tolerance, and the risk for dependence with chronic use [11]. Consequently, current drug development efforts focus on developing GABA_A_R PAMs with subtype-selective activity-enhancing efficacy and minimizing activity at subtypes linked with adverse effects [12,13].

Despite the complexity of GABA_A_R, several subtype-selective PAMs have been developed. For example, α1-selective modulators such as zolpidem and zopiclone have been developed as effective sedative-hypnotics [14]. Other examples include α2-/3-/5-preferring compounds such as PF-06372865 [15], AZD6280 [16], AZD7325 [17], SL65.1498 [18], NS11821 [19], L–838,417 [20,21] and its analogs, TPA-023 [22,23], and TPA023B [24,25]. However, many of these compounds failed to progress clinically, due to issues such as poor bioavailability, insufficient subtype selectivity *in vivo*, and/or toxicological concerns. As a result, there remains a significant unmet need for novel GABA_A_R subtype-selective PAMs, particularly those targeting α2/3/5 subunits, which may offer safer and more targeted treatment options for hyperarousal-related neuropsychiatric conditions, such as anxiety disorders. ENX-102 (C21H18F2N5O5P) is a novel phosphate-associated version of the previously reported α2-/3-/5-selective GABA_A_R PAM TPA023B [24]. The pharmacodynamic (PD) characteristics of non-selective and subtype-selective GABA_A_R agonists have been studied in humans using the NeuroCart, a CNS test battery that combines neuropsychological and neurophysiological assessments with quantitative electroencephalography (qEEG) [26]. Non-selective GABA_A_R PAMs such as lorazepam consistently reduce the saccadic peak velocity (SPV), reduce alertness (via the Visual Analogue Scale (VAS)), impair adaptive tracking, and increase body sway [27]. In contrast, α2/3 subtype-selective GABA_A_R PAMs (TPA023, NS11821, AZD6280, PF-06372865) produce comparable SPV reductions at equipotent doses with minimal effects on VAS alertness, adaptive tracking, or body sway [16,18,19,23,28]. Regression analyses confirm the distinct PD profiles of α2-/3-selective to non-selective PAMs [29]. The SPV, VAS alertness, body sway, and adaptive tracking can be considered putative biomarkers for arousal, subjective alertness, postural stability, and sustained attention, respectively. Thus, α2/3 subtype-selective modulation may attenuate arousal while avoiding the sedative and motor-impairing effects seen with non-selective compounds [29].

qEEG offers a temporally precise and translational measure of electrophysiological responses to CNS-agents [26,30]. Non-selective GABA_A_R PAMs (propofol, lorazepam, midazolam) reliably increase β-band power [17,28,31,32,33,34], a biomarker of GABA_A_R-mediated neurotransmission [35]. They also elevate δ-band activity [16,17,28,36,37,38], which is associated with non-rapid eye movement (NREM) sleep [39], sedation, and anesthesia-induced unconsciousness [40,41], and reduce α-band activity via benzodiazepine-site modulation [17,32,33,35,42]. Emerging evidence suggests that α2-/3-selective GABA_A_R PAMs may produce distinct qEEG spectral signatures. In rodent, compounds like TPA023, TP003, AZD6280, AZD7235, and PF-06372865 dose-dependently increased β- and γ-band power [22,43,44]. In humans, NS11821, AZD6280, and PF-06372865 consistently increased β power and decreased δ and θ power following a single dose [15,16,19,28], unlike lorazepam, which increased both β and δ power in humans and rodents [15,16,17]. These findings suggest that α2-/3-selective PAMs, following single doses, can be distinguished from non-selective PAMs by a qEEG spectral profile characterized by increased β power (and possibly γ), reduced δ and θ power, and variable α activity [35,45]. Together, this PD signature may reflect anxiolytic efficacy without sedation, offering a promising therapeutic profile that is distinct from that of benzodiazepines.

The current study reports the preclinical and clinical pharmacology of ENX-102, across doses predicted to achieve >80% receptor occupancy at α2-/3 subunit-containing GABA_A_Rs. In rodents, sedative and anxiolytic-like effects were investigated following acute and sub-chronic oral dosing. In healthy human volunteers, CNS biomarkers, observed using the NeuroCart, were employed to characterize PD effects following single and repeated dosing over 12 days. Furthermore, qEEG spectral changes in both rodents and humans were also examined to support translational alignment. Finally, pharmacokinetics and safety were also evaluated in healthy human volunteers.

## 2. Materials and Methods

### 2.1. In Vitro Experiments

ENX-102 was evaluated for positive allosteric modulation against four GABA_A_ subtypes (GABA_A_ α1β3γ2L, α2β3γ2L, α3β3γ2L, and α5β3γ2L) using the SyncroPatch automated patch-clamp platform (SyncroPatch 384i for data recording, Nanion Technologies GmBH, Munic, Germany). Assays were conducted at room temperature in PAM mode across 10 concentrations (0.01 nM, 0.1 nM, 1 nM, 3 nM, 10 nM, 30 nM, 100 nM, 300 nM, 1 μM, 10 μM), with each condition tested in duplicate. GABA was applied at EC_5–20_ levels (5–20% of maximal response). Diazepam served as positive control (0.01, 0.1, 1, 10, 100μM) and 0.2% DMSO as a negative control. Modulatory activity was expressed as the percentage of activation relative to the GABA-alone condition, and was calculated using the following equation: %activation = [(Icomp/Icontrol) − 1] × 100, where Icomp is the current amplitude in the presence of both ENX-102 and GABA and Icontrol is the current amplitude in the presence of GABA alone.

### 2.2. In Vivo Experiments in Rats

#### 2.2.1. Elevated Plus Maze

Adult male SD rats from Envigo (Indianapolis, IN, USA) were used to assess anxiety-like behavior via the elevated plus maze (EPM). The EPM relies on rats’ instinct to explore while avoiding open areas; increased time spent in the open arms is interpreted as reduced anxiety following acute dosing [46,47]. Rats (~260–300 g prior to treatment) were randomly assigned to one of five treatment groups: vehicle, chlordiazepoxide (5 mg/kg, intraperitoneally (i.p.), positive control), or ENX-102 (0.1, 0.3, or 1.0 mg/kg, per os (p.o.)). Each group consisted of 10 rats, a sample size that was selected to ensure adequate statistical power. ENX-102 was formulated in 0.5% Tylose MH 300 solution and administered at a dose volume of 1.0 mL/kg; chlordiazepoxide was dissolved in water and administered at a dose volume. Behavioral testing was conducted 30 min post-dose for chlordiazepoxide and at 1- and 24-h post-dose for ENX-102 under acute (single dose) conditions. For sub-chronic testing, ENX-102 was administered once daily for 14 days, with EPM testing being conducted on the final day. Due to the limited reliability of the EPM in detecting anxiolytic effects under sub-chronic benzodiazepine treatment [48,49,50], chlordiazepoxide was intentionally excluded as a positive control in this condition. At the designated time post-treatment, each rat was placed in the center of the EPM, facing an open arm, for a 5-min test session. Movement metrics including distance traveled, time spent in each arm, and entries into each arm were automatically recorded by the computer. The experiment remained blinded throughout the active testing period to minimize bias. Unblinding was performed only after all testing was completed. Additional methodological details are provided in Appendix A.1.

#### 2.2.2. Quantitative EEG

**Figure 1:**
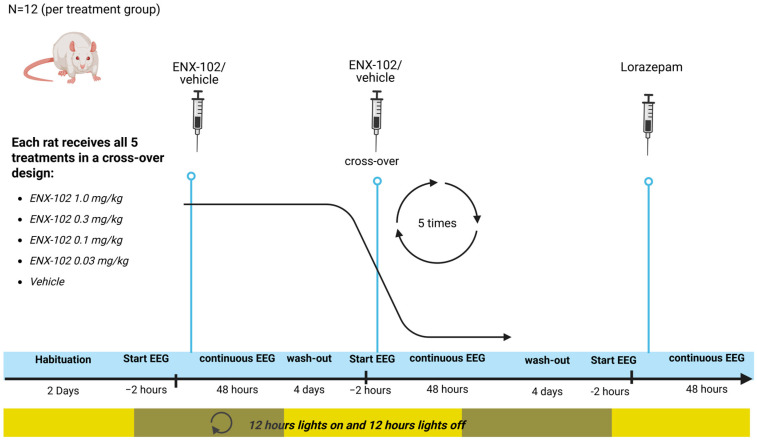
Schematic representation of the qEEG recordings in rats. Twelve rats were used in a cross-over design to assess vehicle and four dose levels of ENX-102. Each dose was followed by a four-day wash-out period. Dosing and EEG recordings were performed on a Monday and Friday schedule, starting during the lights-off period, with dosing occurring 4 h after lights were turned off. EEG recordings began 2 h before dosing and continued for 48 h post-dose. After completion of ENX-102 dosing, all rats (N = 12) received a single dose of lorazepam and EEG was recorded. All animals were habituated to dosing (vehicle dosing) for two days before any data collection took place. Recordings were started 2 h prior to compound administration and recorded continuously for 48 h after administration.

Young adult male Sprague Dawley (SD) rats (~275–325 g on arrival) from Envigo (Indianapolis, IN, USA) were used in this study (rats used for qEEG recordings were distinct from those used in the EPM experiments)**.** A sample size of 10–12 animals per group was determined to provide adequate statistical power. Rats were implanted with electrodes, and EEG was recorded wirelessly while they remained in their home cages. Vehicle and 4 doses of ENX-102 were assessed in a cross-over design to ensure N = 12 rats were included in each treatment group, with a four-day washout. ENX-102 and vehicle (0.5% Tylose MH 300^®^ solution) were administered p.o. Afterwards, all rats (N = 12) received lorazepam and 1.0 mg/kg, i.p., dissolved in saline. Each rat underwent six separate 48-h continuous EEG recordings, each of which was preceded by a 2-h baseline EEG (Figure 1). A more detailed technical description of the qEEG recordings is given in Appendix A.2.

#### 2.2.3. Sleep Analysis

Sleep parameters in rats were assessed as part of the qEEG recordings following administration of ENX-102 at four doses (0.03, 0.1, 0.3, 1.0 mg/kg) or lorazepam. Sleep stages analyzed included active wake, quiet wake, NREM, and rapid eye movement (REM) sleep. A more detailed technical description of the sleep analysis is given in Appendix A.3. Unblinding occurred after sleep scoring during the creation of the decoder used for data processing and final analysis.

### 2.3. Multiple Ascending Dose (MAD) Study in Healthy Human Participants

#### 2.3.1. Study Design

A randomized, double-blind, placebo-controlled, multiple ascending dose study was conducted at Centre for Human Drug Research (Leiden, The Netherlands). A total of 40 healthy participants—both male and female—were enrolled in the MAD study. The sample size was based on precedents from comparable clinical studies [13,24], providing adequate power to assess safety, tolerability, pharmacokinetics, and pharmacodynamic effects. Participants were admitted to the Centre for Human Drug Research (CHDR) in Leiden, the Netherlands, from day −1 to day 13. Safety, pharmacokinetic (PK), and pharmacodynamic (PD) assessments were performed during this period. Follow-up visits occurred on day 19 and day 26, during which only safety and PK assessments were conducted.

#### 2.3.2. Study Population

Healthy females and males aged 18 to 55 years, with a body mass index of 18 to 35 kg/m^2^, were included. To evaluate eligibility, participants underwent eligibility screening, which included review of their medical and psychiatric history, physical examination, vital sign measurements, an electrocardiogram (ECG), blood chemistry and hematology laboratory tests, and urinalysis. Participants were excluded if they had a clinically significant condition within two years prior to screening that could pose a risk to or interfere with study outcomes (e.g., cardiac, renal, neurologic, gastrointestinal, pulmonary, endocrine, hematologic, immunologic disorders, or malignancy), a current or past psychiatric disorder as defined by Diagnostic and Statistical Manual-5, suicidal ideation within 30 days before screening, or suicidal behavior within the past two years. In addition, participants with a history of convulsions were excluded. Participants were not allowed to have ingested any concomitant medication (excluding hormonal birth control) within 5 half-lives or 30 days (whichever was longer) prior to day 1. In total, ENX-102 was administered across five cohorts, each consisting of eight healthy participants in a 6:2 ratio of active treatment to placebo. This resulted in six participants receiving ENX-102 at each dose level, while the 10 placebo participants across all cohorts were pooled for analysis.

#### 2.3.3. Participant Disposition and Demographics

A total of 104 potential participants underwent screening, of whom 40 (18 female and 22 male) were randomized and completed the study. The consort diagram of randomized participants is shown in Figure S1. The mean (range) age and BMI were 30.5 years (19–52) and 25.2 kg/m^2^ (18.5–34.7), respectively. Find more details on the demographics in Table S2.

#### 2.3.4. Dosing Scheme

All doses were administered once daily as p.o. capsules for 12 days, with a fixed amount of 240 mL water intake in a fasted state (≥10 h fasting). In the current MAD study, dose escalation from 0.5 mg to 1.0 mg, 1.5 mg, 2.0 mg, and 5.0 mg was conducted during dedicated dose-escalation meetings, following blinded review of all available PK, PD, and safety data of each cohort. The starting dose of 0.5 mg ENX-102 and anticipated dose escalation scheme were selected based on physiologically based pharmacokinetic (PBPK) modeling of preclinical PK data, unpublished clinical data from the single ascending dose (SAD) study, and published work with the TPA-023B free base, in combination with available non-clinical toxicology data obtained in animals and converted to a human equivalent dose [12]. The SAD study investigated ENX-102 0.5, 1.0, 1.5, 2.0, 3.0, 5.0, and 10.0 mg, in which the observed T_max_ ranged from 1.3 to 4 h and the elimination half-life ranged from 43 to 73 h.

#### 2.3.5. Safety Assessments

Adverse events (AEs), serious adverse events (SAEs), vital signs, electrocardiogram (ECG), and clinical laboratory tests were assessed throughout the study. The definition of an SAE used in this study is provided in Appendix B. Clinical laboratory tests included urinalysis, hematology, chemistry, and coagulation analysis. Supine blood pressure and heart rate were measured after at least 10 min of rest in supine position; standing blood pressure was measured at 1, 3, and 5 min after rising from the supine position to standing to assess orthostasis (i.e., systolic blood pressure decrease > 20 mmHg or diastolic blood pressure decrease > 10 mmHg). Vital signs were assessed pre-dose and at 1- and 5-h post-dosing on days 1 through 12. In addition, the Modified Observer’s Assessment Alertness/Sedation score (MOAA/S) and Columbia Suicide Severity Rating Scale (C-SSRS) were administered. The MOAA/S, a rater-administered scale ranging from 0 (unresponsive) to 5 (awake), with values of 0 (unresponsive to pain), 1 (responds only to painful stimulus), 2 (responds to mild prodding), 3 (responds to loud/repeated name calling), 4 (lethargic response to name calling), and 5 (awake), was used to assess and quantify sedation [22]. The MOAA/S was recorded on each study day pre-dose and at 0.5, 1, 1.5, 2, 3, 6, 8, and 10 h post-dosing, as well as within 15 min of any reported somnolence or sedation on days 1 through 12. The C-SSRS evaluated emergent suicidal ideation and behavior and was administered pre-dose and on days 1, 6, 12, 19, and 26.

#### 2.3.6. Pharmacokinetic Assessments

Blood samples (6 mL) for PK analysis were drawn on day 1 pre-dose and at 0.5, 1, 1.5, 3, 6, 8, 10, and 12 h post-dosing and on day 12 pre-dose and at 0.5, 1.5, 3, 6, 8, 10, and 12 h post-dosing. Additional samples were taken on every dosing day, approximately 24 h after the previous day’s dose and prior to the subsequent dose, and 24, 72 (day 13), 168 (day 19), and 336 h (day 26) after the last dose on day 12. Assays were performed by Alturas Analytics, Moscow, United States. PK samples were centrifuged within 60 min (2000 G, 10 min, 4 °C), aliquoted in 2.0 mL Sarstedt tubes, and stored at −80 °C within 2 h of collection. ENX-102 analysis was accomplished using a validated high-performance liquid chromatography/liquid chromatography-mass spectrometry/mass spectrometry method. The limit of quantification was 0.500 ng/mL.

#### 2.3.7. Pharmacodynamic (PD) Assessments

##### NeuroCart CNS Test Battery

The NeuroCart CNS test battery was applied for PD profiling of ENX-102. Saccadic peak velocity (SPV), saccadic reaction time, and smooth pursuit eye movements were assessed to evaluate sedation. Adaptive tracking assessed hand-eye coordination and sustained attention, while body sway measured postural stability. Pupil/iris ratio was measured as a marker of autonomic nervous system modulation. The Bond and Lader visual analog scale (VAS) was used to quantify subjective effects commonly associated with a variety of CNS active drugs. The visual verbal learning task (VVLT) was performed, containing 15 words (in 3 alternate versions) covering basic aspects of verbal learning and memory: acquisition, consolidation, storage, and retrieval.

Baseline measurements were obtained one day before the first administration and were repeated twice according to CHDR Standard Operational Procedures. On day 1 and day 12, at expected steady state, NeuroCart was performed at 0.5, 1, 1.5, 3, 6, 8, 10, and 24 h post-dose. The NeuroCart endpoints included the average values of latency (s), degrees per second (degrees/s) of all correct saccades, percentage of time the participant’s eyes smoothly followed the target (%),average performance percentage (%), pupil/iris diameter ratio (mm), anteroposterior movement (mm), VAS alertness (mm) for reaction time, SPV, smooth pursuit, adaptive tracking, pupil size (pupillometry), and body sway, respectively. The endpoints for VVLT were defined as immediate recall: number correct, delayed recall: number correct, and delayed recognition: number correct. See Appendix C for a more detailed technical description of the NeuroCart assessments including the VVLT.

##### Quantitative EEG

Resting-state qEEG recordings were performed with participants in eyes open and eyes closed conditions 0.5, 1.5, 3, 6, 8, 10, and 24 h post-dose. These recordings were conducted in a sequence with other NeuroCart assessments. qEEG recordings were obtained using a 40-channel recording system (Refa-40, TMSi B.V., Oldenzaal, The Netherlands). All signals were sampled at a rate of 1024 Hz and were filtered prior to storage using a first-order recursive high-pass filter with a cut-off frequency of 0.1 Hz to eliminate low-frequency drift. Digital markers were recorded by the amplifier to indicate the start and end of each eye state (eyes open and eyes closed). See Appendix D for a more detailed technical description of the qEEG.

### 2.4. Statistical Analyses

#### 2.4.1. *In Vivo* Experiments in Rats: EPM, qEEG, and Sleep Analysis

Rat movement metrics from the EPM were analyzed using one-way analysis of variance (ANOVA) followed by Tukey’s post hoc test (95% confidence interval (CI); *p* < 0.05). Data were presented as the mean ± standard error of the mean (SEM). Statistical outliers were defined as values ≥ 2 standard deviations from the group mean. Spectral qEEG data were analyzed in MATLAB version 2019 R, with two pre-dose recordings pooled as baseline. Spectral analysis included quantifying raw, relative, and percent change in spectral power across the traditionally defined EEG frequency bands: δ (0.5–3.9 Hz), θ (4–7.9 Hz), α (8–11.9 Hz), β (12–29.9 Hz), low γ (30–49.9 Hz), and high γ (50–100 Hz). The percentage change from baseline was calculated per channel, rat, dose level, and time segment, then averaged by group. Drug effects were evaluated by comparing treatment groups to the vehicle using one-way ANOVA with Dunnett’s test applied to each 60-min interval. For sleep analysis, REM latency was defined as the time between first NREM onset and the first REM episode (TREM–TNREM). Time spent in each sleep state was expressed as a percentage of total time in each sleep state (mean ± SEM). Data were organized by treatment group, sleep state, and time episode and exported to GraphPad PRISM.

#### 2.4.2. MAD in Humans: Safety, PK, and PD Analyses

Safety analyses were performed using descriptive statistics. PK parameters were summarized by dose level and day using descriptive statistics (n, arithmetic mean, SD, coefficient of variation percentage (CV%) for the arithmetic mean, median, minimum, maximum, geometric mean, and CV% for the geometric mean). The maximal concentration (C_max_), time to reach C_max_ (T_max_), concentration immediately prior to dosing during multiple administrations (C_trough_), last quantifiable concentration (C_last_), time of the last quantifiable concentration (T_last_), apparent terminal elimination rate constant (λ_z_), area under the concentration–time curve from time zero to 24 h (AUC_0–24_) and extrapolated to infinity time after dosing (AUC_0-inf_), apparent oral clearance (CL/F), apparent volume of distribution (Vz/F), accumulation ratio (RAUC), and dose-normalized AUC_0–24_, AUC_0-inf_, and C_max_ were derived directly from individual concentration–time profiles using non-compartmental analysis. A steady state was assumed if the C_trough_ values plotted against administration day formed a line with an approximately zero slope, as assessed by visual inspection. Dose proportionality for ENX-102 AUC_0-inf_ (day 1), AUC_0–24_ (day 12), and C_max_ (day 1 and day 12) were examined using a model with fixed effects for the log-dose and time points (day 1 or day 12) and log-dose by day interaction and random participant effect.

For analysis of the NeuroCart CNS test battery assessments, placebo participants from all cohorts were grouped for comparison. Active treatment groups were compared with placebo using mixed-model analysis of covariance (ANCOVA), with treatment, time, and their interaction as fixed factors. Participant was treated as a random factor, with baseline measurements from day −1 as covariates. Kenward–Roger approximation was used to estimate denominator degrees of freedom, and model parameters were determined using restricted maximum likelihood. Contrasts of placebo vs. single doses (0.5–5.0 mg) of ENX-102 were reported on day 1 and day 12 along with 95% CI, least square mean (LSM) estimates, and *p*-values, with no adjustments made for multiple comparisons since PD measures were all considered exploratory in nature. All PD analyses including qEEG were performed using SAS software, version 9.4 or higher.

## 3. Results

### 3.1. In Vitro Experiments

**Figure 2:**
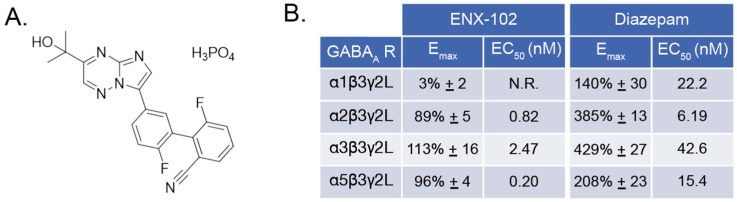
Overview of the chemical structure and *in vitro* pharmacological profile of ENX-102. (**A**) Chemical structure of ENX-102. (**B**) *In vitro* receptor activity of ENX-102 and diazepam at various GABA_A_ receptor subtypes (α1β3γ2L, α2β3γ2L, α3β3γ2L, α5β3γ2L), showing maximum effect (E_max_) and half-maximal effective concentration (EC_50_) values. Data are presented as mean ± SEM. Abbreviations: GABA_A_ R = γ-aminobutyric acid type A receptor; E_max_ = maximum effect; EC_50_ = concentration for half-maximal response; N.R., not reported.

ENX-102 demonstrated selective partial PAM activity at α2β3γ2L, α3β3γ2L, and α5β3γ2L GABAA receptors, with no activity at α1β3γ2L. The E_max_ values were 89% (α2), 113% (α3), and 96% (α5), with corresponding EC_50_ values of 0.82 nM, 2.47 nM, and 0.2 nM (Figure 2B). At α1, ENX-102 showed minimal activation (E_max_ 3%), and no EC_50_ was determined. Diazepam, used as a reference, showed robust PAM activity across all subtypes (Emax 140–429%, EC_50_ 6.2–42.6 nM).

### 3.2. In Vivo Experiments in Rats

#### 3.2.1. Elevated Plus Maze

**Figure 3:**
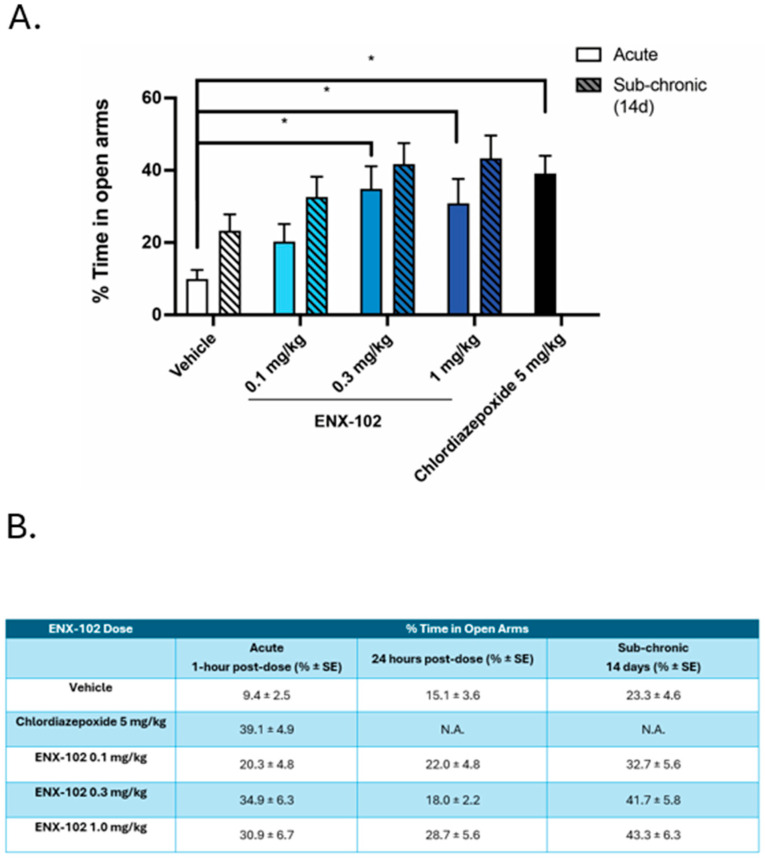
Effects of orally dosed ENX-102 in the rat elevated plus maze (EPM) test. (**A**) ENX-102 was tested in the rat EPM at 0.1, 0.3, and 1 mg/kg dose levels (p.o.) compared with vehicle or the positive control, chlordiazepoxide at 5 mg/kg (i.p.) administered acutely. One hour post-ENX-102 treatment (blue shaded bars) at 0.3 and 1 mg/kg, the percentage of time spent in the open arms of the EPM was significantly increased as compared to vehicle treatment (white bar), and was at a level comparable to that of the positive control chlordiazepoxide (black bar). Data were analyzed by ANOVA followed by post hoc analyses where appropriate. * *p* < 0.05 compared to vehicle. (**B**) Mean percentage of time-spent and standard error (SE) in the open arms at three time points: acute (1 h post-dose), 24 h post-dose, and sub-chronic (after 14 days dosing). N.A. = not applicable.

In the vehicle-treated group, on average, the rats spent less than 9.4% (±2.5 SE) of the time in the open arms of the EPM, while chlordiazepoxide 5.0 mg/kg significantly increased this to 39.1% (±4.9 SE) (Figure 3). Similarly, ENX-102 dose-dependently increased the percentage of time spent in open arms. While the lowest (0.1 mg/kg, corresponding to a human equivalent dose (HED) ~1.0 mg for a 70 kg individual [51]) did not significantly increase the percentage of time spent in open arms, both 0.3 mg/kg (HED~3.0 mg) and 1.0 mg/kg (~10 mg) showed robust anxiolytic-like effects with significantly greater percentages of time spent in open arms (34.9% (±6.3 SE), 30.9% (±6.7 SE)). Notably, the magnitudes of the anxiolytic-like effects for ENX-102 0.3 mg/kg and 1.0 mg/kg were comparable to that of the positive control, chlordiazepoxide (39.1% (±4.9 SE)).

Anxiolytic-like effects of ENX-102 were maintained with sub-chronic treatment, as rats maintained or increased their time spent in the open arms (32.7% (±5.6 SE) at 0.1 mg/kg, 41.7% (±5.8 SE) at 0.3 mg/kg, and 43.3% (±6.3 SE) at 1.0 mg/kg) for up to 14 days of dosing (Figure 3B). However, these differences were not statistically significant compared to vehicle-treated controls. No animals were excluded from this study or analysis.

#### 3.2.2. Quantitative EEG

**Figure 4:**
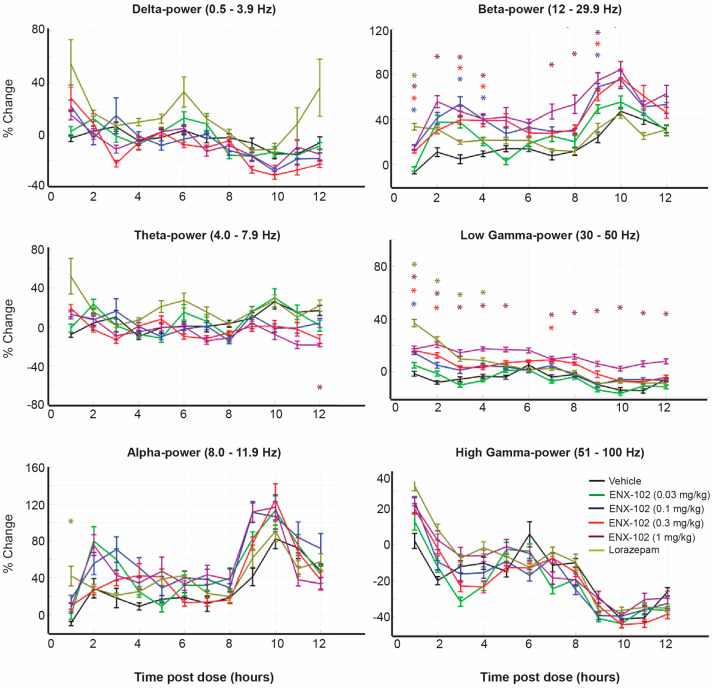
qEEG in rats during quiet wake (0–12 h post-dose)—frontal cortex ENX-12 and lorazepam: Doses tested: vehicle, ENX-102 (0.03, 0.1, 0.3, 1.0 mg/kg), and lorazepam (1.0 mg/kg). Percent change (mean ± SEM) in δ, θ, α, β, low-γ, high-γ bands compared to baseline. Statistical significance indicated with * at different timepoints for each frequency band. Statistically significant increases at T_max_ (1.5–4.5 h) compared to baseline were observed on the β band for ENX-102 0.1, 0.3, and 1.0 mg/kg and in the low-γ band for ENX-102 0.3, 1.0 mg/kg. For lorazepam, statistically significant increases at 1 h post-dose compared to baseline were observed on the β band, α band, and low-γ band. * *p* < 0.05 compared to vehicle.

Compared to baseline, a single oral dose of ENX-102 in the range of 0.1 mg/kg to 1.0 mg/kg (corresponding to a HED of ~1.0 to 10 mg) dose-dependently increased the β-band power in the quiet wake state between 1.5 and 4.5 h post-dose, aligning with its T_max_ in rats. A single dose of lorazepam i.p. at 1.0 mg/kg, with a T_max_ of approximately 8 min [52], showed a significant increase in β-band power at 1 h post-dose (*p* < 0.05; Figure 4). In addition, ENX-102 at 0.3 and 1.0 mg/kg and lorazepam at 1.0 mg/kg significantly increased the low-γ-band power compared to baseline between 1.5 and 4.5 h post-dose and lorazepam at 1 h post-dose, respectively (*p* < 0.05; Figure 4). Neither ENX-102 nor lorazepam significantly altered the lower frequency bands (δ, θ) or high-γ power, while lorazepam increased the α-band power at a single timepoint, 1 h post-dose, compared to baseline. See Figure S2 for spectral changes observed at 2 h post-dose, which especially highlight the dose-dependency of the γ power increase.

Compared to baseline during NREM, ENX-102 0.1 mg/kg to 1.0 mg/kg dose-dependently decreased the δ- and θ-band power, and 0.3 mg/kg to 1.0 mg/kg dose-dependently increased the low-γ-band power at 2, 4, 6, 8, 10, and 12 h post-dose and the high-γ-band power at 2, 3, 5 and 6 h post-dose (Figure S3). Lorazepam decreased the θ power at all time points except 7, 10, 11, and 12 h post-dose and the β power at 3, 4, 5, 6, and 8 h post-dose, which seemed comparable to ENX-102 0.3 mg/kg (*p* < 0.05; Figure S3). ENX-102 did not consistently affect the α or β power in rodents, while lorazepam did not consistently affect the δ, α, or γ power (Figure S3).

These data reveal a dose-dependent EEG spectral signature for ENX-102 that is distinct from that of lorazepam.

#### 3.2.3. Sleep Architecture

**Figure 5:**
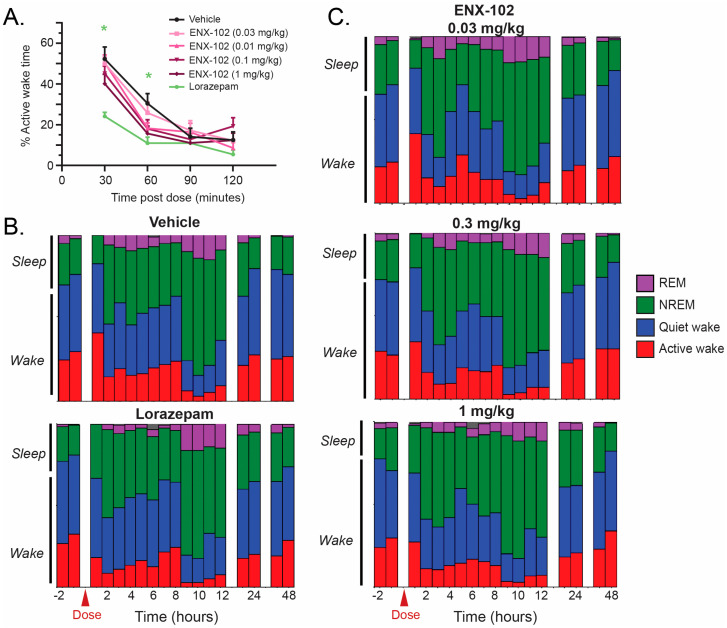
Effect of ENX-102 on sleep architecture in rats. Effect of ENX-102 (at four dose levels) on sleep architecture was examined. (**A**) Data over the first two hours after treatment, with peak plasma levels for ENX-102 and lorazepam, are shown. Lorazepam (green) significantly reduced % time spent in active wake within an hour after administration; ENX-102 (pink) at the different doses tested, 0.01 mg/kg, 0.03 mg/kg, 0.1 mg/kg, or 1 mg/kg, did not significantly impact % time in active wake as compared to vehicle (black)-treated controls. (**B**,**C**) Hypnograms showing proportion of time spent in sleep stages (REM, purple; NREM, green) or awake (quiet wake, blue; active wake, red), for all groups from two hours before dosing up to 48 h after dosing. * *p* < 0.05 compared to vehicle (ANOVA, Dunnett’s test). Abbreviations: REM = rapid eye movement; NREM = non-rapid eye movement.

Lorazepam significantly reduced the percentage of time spent in active wake as compared to vehicle-treated controls (*p* < 0.05; Figure 5A). In contrast, ENX-102 did not significantly impact the percentage of time spent in active wake at any of the doses within the first two hours post-dose, which coincided with the ENX-102 T_max_ in rats approximately 1.5 to 4.5 h post-dose. When examined over a time period of up to 24 and 48 h, hypnograms reveal no significant differences in the proportion of time spent in REM or NREM sleep stages and active or quiet wake stages in ENX-102-treated groups (Figure 5B,C). Based on the onset of REM sleep, the vehicle-treated rats required ~40 min to go into NREM sleep, which was significantly reduced by lorazepam (~20 min), whereas ENX-102 did not impact the latency to onset of NREM sleep at any of the doses tested (*p* < 0.05; Figure S4A).

Together, these data show that ENX-102 did not cause sedation or increase the propensity to sleep in rats, even at the highest dose tested (1.0 mg/kg), corresponding to a HED of ~10.0 mg for a 60 kg human.

#### 3.2.4. Pharmacokinetics

Average C_max_ and AUC_0–t_ increased dose-dependently over the 0.1 to 3.2 mg/kg dose range (C_max_ 35 to 1505 ng/mL; HED = ~3 to 30 mg), with an average T_max_ and T_1/2_ ranging from 1.5 to 4.5 h and 15.5 to 19.1 h, respectively (Table S2).

### 3.3. Multiple Ascending Dose (MAD) Study in Healthy Human Participants

#### 3.3.1. Safety

AE data are provided in Tables S3 and S4. Over the 12-day dosing period, a total of 408 post-dose AEs were reported, of which 354 (87%) occurred in 30 (100%) participants receiving ENX-102 and 54 (13%) occurred in 9 (90%) participants receiving placebo. The majority of reported AEs (402/408 = 98.5%) in most participants (34/40 = 85%) were mild in intensity and all were self-limiting (data on file). No serious or severe AEs occurred. Five participants experienced moderate AEs, accounting for one event each of gastroenteritis (reported 6 days after final dosing on day 12), headache (reported 9 days after final dosing on day 12), balance disorder (reported on day 1 of dosing), and muscle pain (reported on day 7 of dosing) and two events of somnolence (reported on day 1 and day 6 of dosing in two different participants). These six moderate AEs represent approximately 1.5% of all post-dose AEs (6 of 408). There was no clear dose-dependent increase in incidence of AEs. On day 1, the ENX-102-treated groups reported relatively more AEs than the placebo-treated group. On Day 12, the number of AEs reported was generally lower than on day 1, and they were approximately equally distributed across ENX-102 and placebo groups.

**Figure 6:**
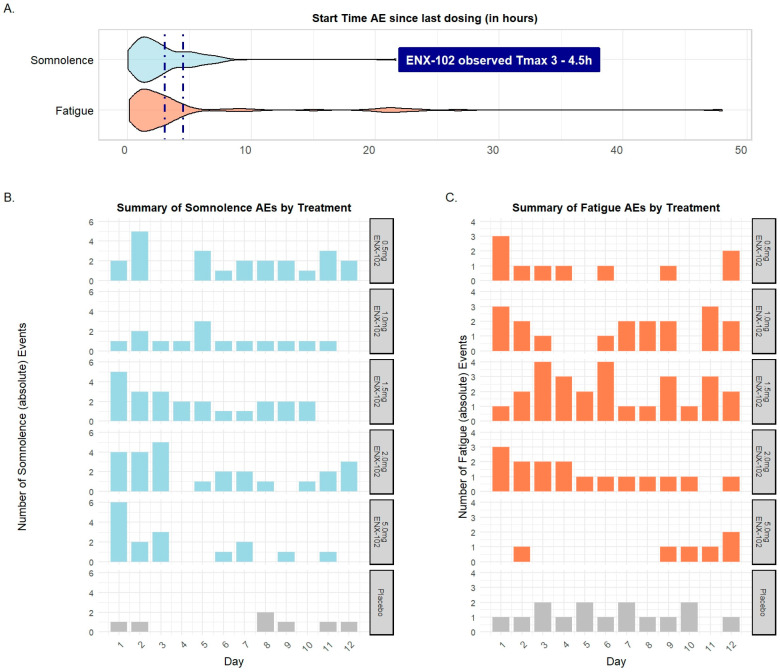
Summary of the most commonly reported adverse events (AEs), somnolence and fatigue, in the human multiple ascending dose (MAD) study. (**A**) Violin plots showing the distribution of onset times of fatigue and somnolence after dosing, relative to the median T_max_ window (3–4.5 h) for ENX-102, across days 1–12. (**B**) Absolute number of somnolence AEs for placebo and ENX-102 0.5, 1.0, 1.5, 2.0, and 5.0 mg per dosing day. (**C**) Absolute number of fatigue AEs for placebo and ENX-102 0.5, 1.0, 1.5, 2.0, and 5.0 mg per dosing day.

The most commonly reported AEs over the 12-day dosing period were somnolence and fatigue in both the ENX-102- and placebo-treated groups. In the ENX-102-treated group, somnolence and fatigue accounted for 28.5% (101/354) and 22.5% (80/354) of the total AEs, respectively. Similarly, in the placebo-treated group, somnolence and fatigue were the most commonly reported AEs, with somnolence occurring in 13% (7/54) and fatigue occurring in 27.8% (15/54) of participants, respectively. The incidence of somnolence for ENX-102 0.5 mg, 1.5 mg, 2.0 mg, and 5.0 mg was lower on day 12 compared to day 1 (Table S3, Figure 6B,C). Somnolence and fatigue occurred at a median time of 2.5 h (range: 2.25–23 h for somnolence and 2.38 to 7 days) after administration of ENX-102 (Figure 6A). No clinically relevant changes were observed in laboratory evaluations, vital signs, or ECGs. None of the participants reported suicidal ideation or behavior on the C-SSRS. No deaths or serious adverse events (SAEs) occurred.

These findings demonstrate that repeated dosing of ENX-102 is safe and well tolerated, producing AEs consistent with GABAergic pharmacology, although, importantly, in the absence of sedation, and no evident dose-related increase in somnolence or fatigue was observed. Finally, the occurrence of fatigue and somnolence appeared to coincide with peak plasma exposures and attenuated after repeat administrations.

#### 3.3.2. Pharmacokinetics (PK)

**Figure 7:**
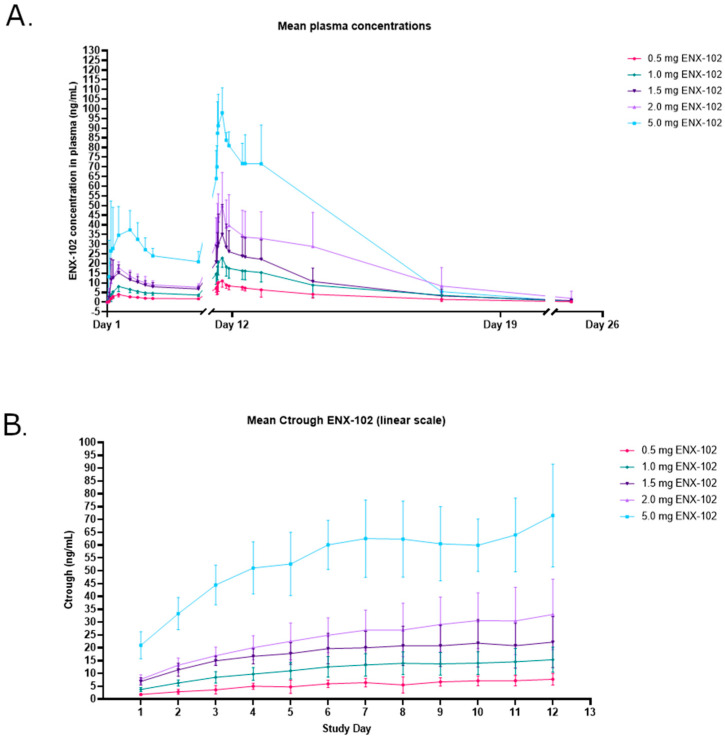
Pharmacokinetic profile of ENX-102. (**A**) Mean plasma concentrations of ENX-102. Mean plasma ENX-102 concentrations in ng/mL for doses of 0.5 mg, 1.0 mg, 1.5 mg, 2.0 mg, and 5.0 mg. (**B**) Mean C_trough_ day 1 to day 12 (linear scale). Mean C_trough_ in ng/mL with standard deviation for doses of 0.5 mg, 1.0 mg, 1.5 mg, 2.0 mg, and 5.0 mg.

ENX-102 was well-absorbed following oral administration, with peak plasma concentrations being reached within 3–4.5 h, followed by a bi-exponential decrease, with a mean terminal half-life between 39 and 66 h (Figure 7A, Table S5). Table S5 summarizes the PK results on days 1 and 12 after the multiple doses of ENX-102 and shows that mean C_max_ and AUC_0–24_ generally increased dose- and concentration-proportionally over the investigated dose range. On day 1, the C_max_ ranged from 4.6 to 49.7 ng/mL, while, on day 12, it varied from 11.7 to 99.1 ng/mL. The mean half-life (T_1/2_) behaved as anticipated for all dose levels, except for the 5.0 mg group, which exhibited a relatively shorter half-life. Accumulation of ENX-102 at plasma steady-state concentrations (Css) was evident, with a mean accumulation ratio (R_ac_) ranging from 3.0 to 4.0. For ENX-102 < 2.0 mg, the C_trough_ slope stabilized after days 6 to 12 days of dosing (Figure 7B), indicating that a steady state was achieved. However, the 2.0 mg and 5.0 mg doses did not show the same C_trough_ slope stabilization as lower doses, suggesting the emergence of non-linear PK at higher doses (Figure 7B). The CV% ranged between 30 and 40% for the T_1/2_, C_max_, and AUC across the investigated dose range.

Overall, ENX-102 PK demonstrated dose-proportionality, relatively low variability, and a long half-life, with steady-state plasma concentrations being reached by day 12, which make it well-suited for chronic treatment.

#### 3.3.3. Pharmacodynamic (PD) Effects

**Figure 8:**
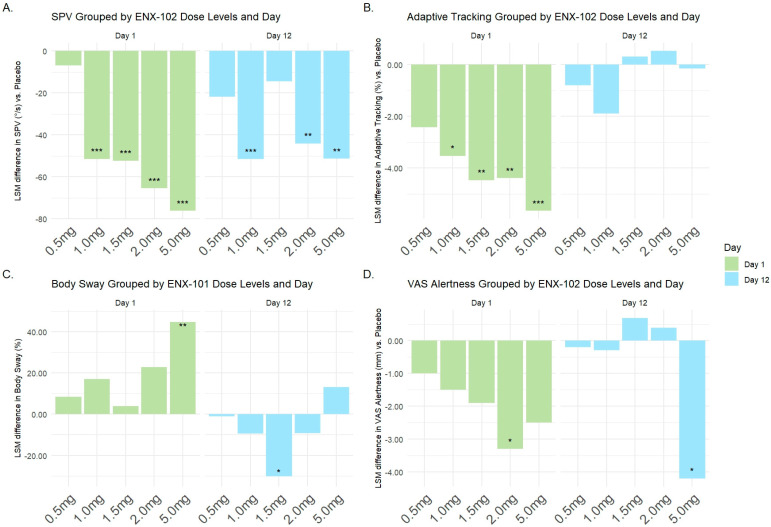
Least square mean (LSM) difference in pharmacodynamic effects on the different NeuroCart assessments between ENX-102 dose groups and placebo after single dose (day 1) and multiple dose (day 12). (**A**) Saccadic peak velocity (SPV) (%/s). (**B**) Adaptive tracking (%) by dose group and day, and panel. (**C**) Body sway (%) by dose group and day, and panel. (**D**) Visual Analogue Scale (VAS) alertness by dose group and day. Statistical significance is indicated by: *** *p* < 0.001; ** *p* < 0.01; * *p* < 0.05 baseline corrected compared with placebo.

A detailed summary of the PD results can be found in Table S6. Figure 8 and Figure S5 provide a visual overview of the most informative PD effects of ENX-102 across dose levels and time points. Compared with placebo, the ENX-102 doses ≥ 1.0 mg statistically significantly decreased the SPV on day 1 following a single dose, with estimated differences of −51.46 (95% CI: −80.31 to −22.60), −52.40 (95% CI: −80.83 to −23.97), −65.51 (95% CI: −94.43 to −36.59), and −76.22 (95% CI: −106.35 to −46.09) for the 1.0 mg, 1.5 mg, 2.0 mg, and 5.0 mg doses, respectively (all *p* < 0.001; Table S6). This effect was sustained on day 12, with estimated differences compared with placebo of −51.59 (95% CI: −80.44 to −22.73; *p* < 0.001), −44.11 (95% CI: −73.03 to −15.19; *p* = 0.004), and 5.0 mg −51.39 (95% CI: −81.13 to −21.65; *p* = 0.001) for the 1.0 mg, 2.0 mg, and 5.0 mg doses, respectively (Table S6). Moreover, the SPV reductions for ENX-102 1.0 mg, 2.0 mg and 5.0 mg did not return to baseline by 24 h post-dose on either day 1 or day 12, and the SPV remained reduced prior to the last dose on day 12 (Figure S5A and Figure 8A). Decreases in SPV generally emerged approximately 0.5 h post-dose and reached a peak between 1.5 and 3 h post-dosing on both day 1 and day 12. ENX-102 5.0 mg was the exception, as the peak SPV reductions appeared relatively later, at 6 h post-dose (Figure S5A).

In contrast to the SPV, ENX-102 doses ≥ 1.0 mg statistically significantly decreased adaptive tracking compared with placebo on day 1 following a single dose, with estimated differences of −3.532 (95% CI: −6.382 to −0.682; *p* = 0.0164), −4.470 (95% CI: −7.483 to −1.456; *p* = 0.0046), −4.399 (95% CI −7.310 to −1.489; *p* = 0.0040), and −5.660 (95% CI −8.530 to −2.791; *p* < 0.001) for the 1.0 mg, 1.5 mg, 2.0 mg, and 5.0 mg doses, respectively (Table S6). However, the effect returned to baseline by 12 h post-dose and had disappeared by day 12 (Figure 8; Figure S5B; Table S6). In addition, only ENX-102 5.0 mg statistically significantly increased the SRT and body sway compared to placebo on day 1 following a single dose, with estimated differences of 0.0198 (95% CI: 0.0032 to 0.0365); *p* = 0.0206) for SRT and 44.8 (95% CI: 10.9 to 89.0; *p* = 0.0077) for body sway. On day 12, body sway was statistically significantly decreased for ENX-102 1.5 mg, with an estimated difference of −30.2 (95% CI: −46.8 to −8.4; *p* = 0.0107), but not at any other dose, and no effects were present at any dose for SRT (Figure S5F; Table S6). Furthermore, smooth pursuit eye movements, the pupil/iris ratio, and VAS alertness were comparable to placebo and relatively inconsistent across the investigated dose range on day 1. On day 12, VAS alertness was statistically significantly decreased for ENX-102 5.0 mg, with an estimated difference compared with placebo of −4.2 (95% CI: −7.5 to −0.9); *p* < 0.0141), but not at any other dose (Figure 8D; Table S6). No effects were present at any dose for smooth pursuit eye movements or the pupil/iris ratio (Table S6).

VVLT performance was comparable to placebo across the investigated dose range on day 1, except for ENX-102 5.0 mg, which showed statistically significant decreases in immediate word recall, delayed word recall, and delayed word recognition, with estimated effects compared with placebo of −3.8 (95% CI: −6.8 to −0.9; *p* = 0.0119), −4.9 (95% CI: −9.0 to −0.8; *p* = 0.0189), and −3.0 (95% CI: −6.0 to −0.0; *p* = 0.0485), respectively. On day 12, neither immediate nor delayed word recall was affected, while delayed word recognition exhibited inconsistent effects across doses with no dose relationship (Table S6).

Together, these data suggest that ENX-102 sustained reduced arousal following multiple dosing, as indicated by a decreased SPV, while undesired CNS effects, such as impairments in sustained attention, memory dysfunction, postural stability, or subjective alertness, diminished over time, were absent by day 12, or presented an inconsistent pattern that would not suggest clinical meaningfulness.

#### 3.3.4. Quantitative EEG

**Figure 9:**
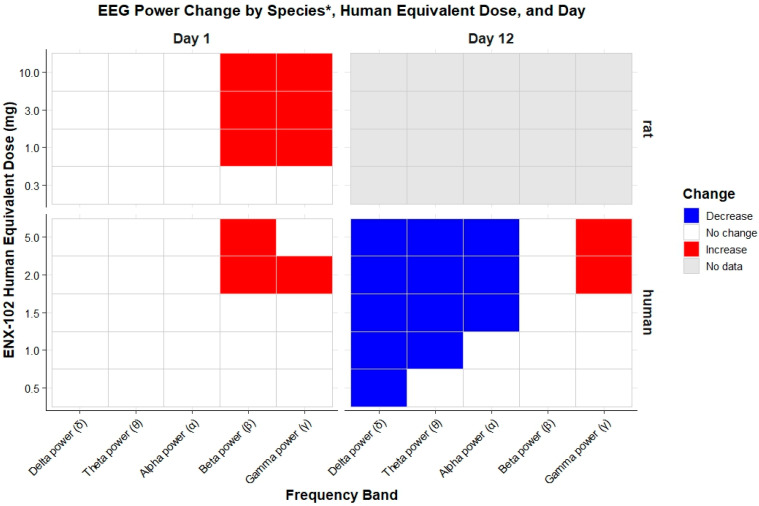
Direction of EEG power change by species, human equivalent dose (HED), and day. (* frontal cortex in rats and Fz-Cz in humans) *Y*-axis shows ENX-102 dose levels: in rats (0.03, 0.1, 0.3, 1.0 mg/kg; approximately equivalent to 0.3, 1.0, 3.0, and 10 mg HED for a 60 kg human, respectively) and in humans (0.5, 1.0, 1.5, 2.0, 5.0 mg). *X*-axis displays EEG power spectral bands (δ, θ, α, β, γ). Red indicates a statistically significant increase in EEG power (vs. placebo in humans, vs. baseline in rats); blue indicates a significant decrease (vs. placebo in humans); white indicates no significant change; grey denotes no data available for rats on day 12. On day 1, significantly increased β and γ power is observed at higher doses (2.0 mg in humans, 1.0 mg/kg in rats). On day 12 in humans, a significant decrease in δ, θ, and α power was observed starting at 0.5 mg, with a significant increase in γ power only at higher doses (2.0 and 5.0 mg).

A detailed summary of qEEG results can be found in Table S7. Figure 9 illustrates the direction of EEG power changes induced by ENX-102 in both humans and rats, measured under eyes-closed conditions in the frontal cortex in rats and the fronto-central region in humans. On day 1, compared with placebo and under eyes-closed conditions, ENX-102 1.5 mg statistically significantly reduced both α and γ in the parieto-occipital region (Pz-O1), with estimated differences of −36.0 (95% CI: −57.1 to −4.7; *p* = 0.0290) and −42.0 (95% CI: −60.2 to −15.4; *p* = 0.0056), respectively. ENX-102 2.0 mg and 5.0 mg statistically significantly increased the β power in the fronto-central region (Fz-Cz), with estimated differences of 74.9 (95% CI: 29.5 to 136.1; *p* = 0.0005) and 71.3 (95% CI: 26.9 to 131.4; *p* = 0.0008), respectively, and reduced the γ power in the parieto-occipital region (Pz-O1), with estimated differences of −34.8 (95% CI: −55.2 to −5.0; *p* = 0.0266) and −36.3 (95% CI: −56.3 to −7.3; *p* = 0.0197), respectively (Table S7). On day 12, under eyes-closed conditions, ENX-102 at all dose ranges statistically significantly reduced the δ power in the fronto-central region compared to placebo. The estimated differences range from −29.5 (95% CI: −48.9 to −2.7; *p* = 0.0340) to −52.4 (95% CI: −65.9 to −33.4; *p* < 0.0001). In addition, the parieto-occipital regions showed statistically significant reductions in δ power at ENX-102 doses of 1.0 mg and above. Furthermore, ENX-102 at doses ≥ 1.0 mg statistically significantly reduced the θ power in the fronto-temporal region, with estimated differences ranging from −49.2 (95% CI: −64.9 to −26.6; *p* = 0.0006) to −72.9 (CI −81.1; −61.2; *p* < 0.0001). ENX-102 1.5 and 2.0 mg also led to statistically significant reductions in α power across all regions compared to placebo, with estimated differences as follows: Fz-Cz: −56.4 (95% CI: −73.5 to 28.2; *p* = 0.0018) and −58.5 (95% CI: −74.8 to −31.9; *p* = 0.0009), Pz-O1: −49.5 (95% CI: −66.1 to −24.8; *p* = 0.0013) and −46.1 (95% CI: −63.8 to −19.7; *p* = 0.0032), and Pz-O2: −48.0 (95% CI: −64.4 to −24.2; *p* = 0.011) and −53.4 (95% CI: −68.0 to −32.2; *p* = 0.0002). Additionally, ENX-102 at 2.0 mg statistically significantly increased the γ power in the fronto-central region compared to placebo, with an estimated difference of 36.1 (95% CI: 2.0 to 81.7; *p* = 0.0366). Finally, ENX-102 5.0 mg not only statistically significantly reduced the δ, θ, and α power across all regions, but also increased and decreased the γ power at Fz-Cz and Pz-O2, respectively (*p* < 0.05 for all comparisons; see Table S7 for detailed estimates).

In summary, the most prominent observed effect following single doses of ENX-102 was increased β power, whereas the most consistent steady-state effects following multiple doses included decreased δ, θ, and α power, across the investigated dose range of 0.5 to 5.0 mg.

## 4. Discussion

The current report summarizes the preclinical and clinical pharmacological characteristics of ENX-102, a novel GABA_A_R α2-/3-/5-selective PAM that is currently under development for anxiety disorders. *In vitro* studies demonstrated that ENX-102 acts as a partial PAM selectively at ⍺2/3/5 subunit-containing GABA_A_Rs and a functional antagonist at ⍺1 subunit-containing GABA_A_Rs. This is a distinguishing profile from diazepam, a non-selective GABA_A_ PAM, as diazepam additionally activates α1 subunit-containing GABA_A_Rs associated with unwanted side-effects. In humans, ENX-102 dose-dependently decreased the saccadic peak velocity (SPV), a pharmacological biomarker that reflects CNS arousal and is therefore a putative surrogate for anxiolysis, which was sustained at steady-state plasma exposures at doses ≥ 1.0 mg, without any indication for CNS suppression, including sedation. These findings are consistent with preclinical data in rodents, demonstrating sustained anxiolytic-like effects in rats at doses ≥ 0.3 mg/kg (human equivalent dose (HED) ~3 mg (Figure 3)) that were devoid of sedative effects up to 1.0 mg/kg (HED ~ 10 mg) (Figure 9). Moreover, resting state qEEG revealed a distinct spectral power profile indicative of GABA_A_R subtype selectivity in both rodents and humans. Finally, ENX-102 exhibited favorable safety and pharmacokinetic (PK) profiles in humans, showing good tolerability and appropriate drug exposure with multiple dosing, which supports its continued clinical development.

The safety profile of ENX-102 in humans appears favorable, particularly compared to non-selective GABA_A_R PAMs, which are often associated with sedation and adverse events related to CNS suppression following single, subchronic, and/or multiple dosing [11]. Notably, no sedation-related AEs were reported in the human MAD study, and although mild somnolence and fatigue were reported, these effects did not demonstrate dose-dependence. Moreover, the occurrence of somnolence and fatigue was transient, associated with plasma C_max_, and most pronounced on day 1, yet it was comparable with placebo by day 12 following repeated daily dosing. These findings are consistent with ENX-102’s lack of activity or antagonism at the GABA_A_R α1 subunit, which is typically associated with non-selective GABA_A_-PAM-mediated sedative effects. Considering that somnolence appeared to be temporally associated with peak plasma concentrations, evening dosing may mitigate the occurrence of somnolence and fatigue when initiating dosing in future studies.

In humans, the PK profile demonstrated dose-proportionality and a relatively long half-life, which makes ENX-102 suited for chronic treatment. With once-daily dosing, a steady state was achieved between 6 and 12 days, with the T_max_ and T_1/2_ ranging from 3.0 to 4.5 h and 39 to 66 h, respectively, and the C_max_ and AUC_0–24_ increasing proportionally with the dose. Accumulation was evident up to 6 days of dosing, due to the long half-life, with no additional accumulation once a steady state was reached. The intersubject PK variability was minimal. Beyond its favorable safety profile, ENX-102 exhibits a pharmacokinetic (PK) profile that supports once-daily oral dosing in future trials.

On day 1 in humans, ENX-102 ≥ 1.0 mg not only reduced SPV dose-dependently but also reduced adaptive tracking and/or VAS alertness, while it increased SRT and body sway. In contrast to day 1, however, at steady-state exposures on day 12, reductions in SPV were sustained for ENX-102 1.0 mg, 2.0 mg, and 5.0 mg, while adaptive tracking, VAS alertness, and/or body sway remained unaffected for ENX-102 1.0 mg and 2.0 mg. This suggests no tolerance to the decrease in arousal and potential anxiolytic effects for ENX-102, whereas potentially adverse CNS side effects such as decreased sustained attention, subjective alertness, and postural stability were well tolerated with repeated administration. At a higher, supratherapeutic dose, the VAS alertness remained reduced at 5.0 mg. Furthermore, SPV reductions were sustained beyond the attenuation of adaptive tracking, VAS alertness, and body sway effects for ENX-102 ≥ 1.0 mg on day 1, and similarly at steady-state exposures on day 12. SPV reductions were sustained up to 24 h after the final dose for ENX-102 1.0 mg, 2.0 mg, and 5.0 mg. In fact, the mean SPV decreases of −40 to −50 degrees per second for ENX-102 1.0 mg and 2.0 mg on day 12 were comparable to those previously demonstrated by a single dose of lorazepam 2.0 mg administered orally [15,16,18,23]. Single doses of ENX-102 therefore effectively reduced arousal and, in parallel, also undesirably decreased sustained attention, subjective alertness, and postural stability, though to a lesser degree than lorazepam 2.0 mg. The reduction in adaptive tracking, VAS alertness, and body sway following a single dose of ENX-102 were also less pronounced than those inducted by lorazepam 2.0 mg [16,18,23].

Consistent with the related AEs of somnolence and fatigue, the transient undesired PD effects are hypothesized to result from an initial supraphysiological activation of GABA-A-R α2/3 subunits, leading to a rapid increase in neural inhibition (initial GABA burst) at doses of 1.5 mg and higher. These effects are not attributed to loss of subtype selectivity, considering ENX-102’s functional antagonism at α1 subunits. At steady-state exposures, however, reduced arousal was robustly sustained for ENX-102 1.0 mg and 2.0 mg, in the absence of deleterious effects on sustained attention, subjective alertness, and/or psychomotor function. Arguably, this is further supported by preclinical data, considering that, in the elevated plus maze (EPM), a well-established rodent model of anxiety, ENX-102 demonstrated anxiolytic-like effects at doses ≥ 0.3 mg/kg (human equivalent dose—HED~3 mg) which were comparable to those of chlordiazepoxide, but no sedative effects were observed up to 1.0 mg/kg (HED~10 mg). These results indicate that ENX-102 1.0 mg to 2.0 mg may effectively sustain reduced arousal over time in the absence of CNS-depressant effects in humans, which was confirmed by the absence of clinical sedation and stable MOAA/S scores from day 1 to day 12 at all dose levels. This is further supported by preclinical data indicating that, at the highest tested dose (HED~10 mg), ENX-102 did not reduce the active wake time in rats, unlike lorazepam. Finally, ENX-102 did not negatively impact memory function in humans, neither following a single dose nor at steady-state exposures. Altogether, these findings align with ENX-102’s lack of α1 subunit activity and behavioral effects in preclinical species, differentiating it from non-selective GABA_A_R PAMs [17] and supporting ENX-102’s potential sustained anxiolytic efficacy without sedation or cognitive impairment, as opposed to non-selective GABA_A_R PAMs.

SPV reductions following single doses on day 1 were larger than those demonstrated at steady-state exposures on day 12 for ENX-102 1.5 mg and higher. For instance, ENX-102 at 2.0 mg and 5.0 mg reduced the SPV in the range of −65.5 to −76.2 degrees per second following single doses, while, at steady-state exposures, ENX-102 2.0 mg and 5.0 mg reduced the SPV to −44.1 and −51.4 degrees per second, respectively. However, the reduction from day 1 to day 12 is more consistent with transient effects from an initial burst of GABA on day 1 rather than a true loss of function over time given that the magnitude of SPV decrease on day 12 was still in the range of an acute anxiolytic dose of lorazepam 2.0 mg. It is well established that tolerance develops at differential rates to different behavioral effects of GABA_A_R PAMs. For example, in contrast to the development of tolerance for somnolence and sedative effects of non-selective GABA_A_R PAMs, tolerance for anxiolytic effects is believed to develop more gradually, to develop partially, or to not occur at all, even following prolonged treatment [53]. Similarly, patients with panic disorder and generalized anxiety disorder demonstrated sustained anxiolytic efficacy that did not diminish with long-term alprazolam or diazepam treatment [53]. Notably, in the MAD study, doses associated with decreased adaptive tracking and VAS alertness and/or increased body sway on both days 1 and 12 were not only associated with relatively greater SPV reductions, but also AEs such as somnolence. Thus, SPV changes following single doses may be conflated by coinciding impairments in attention, alertness, and postural stability on day 1, rather than less sizeable reductions in arousal on day 12 resulting from tolerance.

Resting-state qEEG findings in both humans and rodents seem to indicate an ENX-102 spectral power signature that was not only distinct from non-selective GABA_A_R PAMs [44], but also consistent with other α2-/3-selective PAMs [15,16,19,22,43,44]. In both species, single-dose ENX-102 increased the β and, to a certain extent, albeit less consistently, γ power at comparable plasma exposures, as the C_max_ ranged from ~20 ng/mL to ~50 ng/mL (1.5 mg to 5.0 mg) and 35 ng/mL to 362 ng/mL (HED of 1.0 to 10 mg) in humans and rats, respectively, on day 1, while lorazepam, in contrast, has been reported to increase β and δ activity while decreasing the α power in humans [15,16,17]. In rats, changes in θ band activity have been generally inconsistent [35] and could not readily be distinguished from δ band activity [32,33], while the data on changes in γ band activity are currently too limited to allow for adequate interpretation [17,54]. However, at steady-state plasma exposures for ENX-102 in humans, the β power was unaffected and the γ power remained inconsistent, while, most importantly, decreases in δ, θ, and α power were evident, which was reflective of the single-dose qEEG signatures previously reported for the GABA_A_R α2/3-selective PAMs NS11821, AZD6280, and PF-06372865 [15,16,19]. Although the clinical and/or therapeutic implications of the emergence of decreased δ, θ, and α power coinciding with the disappearance of β enhancement following repeated dosing remain to be elucidated, such changes in power spectrum configuration may be indicative of GABAergic adaptation ensuing from sustained GABA_A_R α2/3/5 target engagement. This interpretation is supported by the observed changes over time in somnolence in absence of sedation, adaptive tracking, VAS alertness, SRT, and body sway in the MAD. Additionally, regional differences in γ activity were evident, with indications for increased activity in frontal cortical regions that contrasted with predominantly decreased activity in parietal-occipital regions. Considering that the frontal cerebral cortex is primarily involved in processes related to cognitive control, whereas the parieto-occipital regions are responsible for sensory integration [55], the observed reductions in parietal-occipital γ activity may indicate modulation of sensory integration by ENX-102, which has been implicated in functional processes relevant to anxiety-associated hyperarousal such as disrupted valence and/or autonomic nervous system hyperactivation [56]. This notion is supported by recent resting-state functional magnetic resonance imaging (fMRI) findings for alprazolam that show network connectivity alterations in individuals with social anxiety disorder, particularly in regions involved in sensory and motor processing- including visual, somatosensory, and motor cortices [57]. Notably, regions particularly involved in somatosensory and visual processing demonstrated considerable functional-anatomical overlap with parietal-occipital areas [58], which provides further plausibility to the notion that modulation of sensory-related activity may contribute to attenuated arousal. While there are ongoing efforts to correlate qEEG changes with fMRI-based functional connectivity, current qEEG hardware lacks the spatial resolution to reliably distinguish changes in γ power originating from different cortical regions [59]. At any rate, these findings arguably implicate ENX-102-mediated parietal-occipital modulation of γ activity in functional changes from which, at least theoretically, anxiety disorders might benefit. Finally, ENX-102 demonstrated rather inconsistent effects on α power across species since, in humans, it was decreased at higher doses (≥1.5 mg), while, in rats, no significant effects were observed. In previous studies, the same seemed to hold true for non-selective GABA_A_R PAMs, since, although these often decreased the α power in humans, such effect was less consistently observed in rodents, as both increases [44] and no changes have previously been found [60]. Similarly, lorazepam increased the α power at a single timepoint in rats in the current study, which possibly resulted from a different α frequency range definition compared to other studies, rather than representing a pharmacological effect. Additionally, qEEG work in rodents has historically focused relatively more on other frequency bands, particularly β bands, which potentially contributes to inconsistent α power findings. Altogether, qEEG demonstrated a distinct spectral power signature in both rodents and humans that was not only similar in both species but also evident at comparable plasma exposures, and, as such, these findings illustrate qEEG to be a potentially valuable and translatable pharmacological biomarker in the early development of subtype-selective GABA_A_R PAMs.

Several strengths and limitations of the current report merit mentioning. While the EPM is primarily suited for assessing acute anxiolytic effects due to habituation, ENX-102’s effect appeared sustained over time. The primary goal was to evaluate ENX-102’s anxiolytic potential alongside chlordiazepoxide; ideally, a follow-up study designed to assess long-term efficacy would strengthen these findings.

In the MAD study, ENX-102’s PD profile was characterized using the NeuroCart test battery, which has previously distinguished the PD profiles of several GABA_A_R α2/3 subtype-selective agonists from non-selective GABA_A_R PAMs in humans [18,24]. However, as all previous studies examined single-dose effects, it did not allow for comparison of ENX-102’s PD profile following multiple-dose administration. Similarly, while single-dose qEEG comparisons between rats and humans were possible, the lack of multiple-dose qEEG data in rats limits the ability to draw definitive translational conclusions about ENX-102’s potential effects at a steady state. ENX-102 1.5 mg revealed rather unexpected findings at steady-state exposures. Unlike the 1.0 mg and 2.0 mg doses, it did not affect the SPV, adaptive tracking, or VAS alertness, although it significantly decreased body sway compared to 1.0 mg and 2.0 mg. This discrepancy appears to stem from individual variability in SPV responses: on day 12, three out of sic participants showed an increase in SPV post-dose at 1.5 mg, while the other three showed a decrease (data on file). Finally, PD effects were demonstrated in rats and healthy, non-anxious, and medication-naive human participants. As dose–response relationships and/or GABA_A_R function may differ in patient populations, such as those with anxiety or epilepsy, dose adjustments may be required in subsequent development phases. Finally, although SPV reductions and qEEG changes were sustained up to day 12 without tolerance apparently developing, the impact of dosing for longer periods of time in patient populations needs further investigation in subsequent clinical trials. The fact that 12 days of dosing resulted in maintained SPV reduction is encouraging and supports further evaluation of ENX-102 for use in anxiety disorders, which are often persistent and require ongoing treatment [61]. Lastly, while SPV may serve as a pharmacological biomarker for GABA_A_R α2/3-mediated changes in arousal, its potential role as a surrogate biomarker for anxiolysis in humans remains to be established.

## 5. Conclusions

The novel GABA_A_R α2-/3-/5-selective PAM ENX-102 was safe and well tolerated in healthy human participants following multiple dosing, demonstrated a PK profile supporting once-daily dosing, and, most importantly, reduced arousal that was sustained and not associated with sedative effects typically associated with non-selective GABA_A_R PAMs targeting α1 subunits. These findings were consistent with both *in vitro* data and *in vivo* behavioral findings at similar plasma exposures in rodents, and, moreover, qEEG revealed a high degree of cross-species consistency and alignment from preclinical models to healthy human participants, illustrating its translational potential as a pharmacological biomarker in the early development of subtype-selective GABA_A_R PAMs. Collectively, these results provide compelling evidence that ENX-102 may have the potential for anxiolytic efficacy without causing sedation, and, more broadly, highlight the value of biomarker-based early clinical characterization of novel CNS-active compounds that are in development for the treatment of neuropsychiatric disease.

## Data Availability

The original contributions presented in this study are included in the article/Supplementary Materials. Further inquiries can be directed to the corresponding author.

## Supplementary Materials

The following supporting information can be downloaded at: https://www.mdpi.com/article/10.3390/cells14201575/s1, Figure S1: Consort diagram of randomized participants.; Figure S2: qEEG signature in rats of ENX-102 at 2 h post-dose.; Figure S3: qEEG in rats at non-rapid eye movement sleep (NREM) (0–12 h post dose).; Figure S4: onset of rapid eye movement (REM), non-rapid eye movement (NREM), and latency for REM for vehicle, ENX-102, and lorazepam in rats; Figure S5: mean (standard pharmacodynamic parameters of the NeuroCart saccadic peak velocity (SPV), adaptive tracking, smooth pursuit eye movements, body sway, VAS alertness) measured at 0.5, 1, 1.5, 3, 6, 8, 10, and 24 h on day 1 and on day 12. Table S1: Demographic and baseline characteristics of study participants by treatment; Table S2: pharmacokinetics for ENX-102 in rats; Table S3: summary of the total number of adverse events (AEs), and the five most commonly reported AEs per treatment group on day 1, day 12, and days 1 through 12; Table S4: summary of number of participants with treatment emergent adverse events (AEs) and number of treatment emergent AEs by system organ class and preferred term; Table S5: pharmacokinetic parameters of ENX-102 0.5 mg, 1.0 mg, 1.5 mg, 2.0 mg, and 5.0 mg at day 1 and day 12; Table S6: central nervous system effects of ENX-102 (0.5 mg, 1.0 mg, 1.5 mg, 2.0 mg, and 5.0 mg) compared to placebo on day 1 and day 12; Table S7: qEEG results of the human multiple ascending dose study eyes open and eyes closed states at day 1 and at day 12.

## Abbreviations

The following abbreviations are used in this manuscript:

α	Alpha
β	Beta
δ	Delta
γ	Gamma
θ	Theta
AE	Adverse Event
ANCOVA	Analysis of Covariance
AUC	Area Under the Curve
BMI	Body Mass Index
CHDR	Centre for Human Drug Research
CNS	Central Nervous System
C_max_	Maximum Plasma Concentration
C_trough_	Trough Plasma Concentration
CV	Coefficent of Variation
EEG	Electroencephalography
EC_50_	Half Maximal Effective Concentration
E_max_	Maximum Effect
EPM	Elevated Plus Maze
Fz-Cz	Fronto-Central EEG electrode region
GABA	Gamma-Aminobutyric Acid
GABA_A_R	GABA_A_ Receptor
HED	Human Equivalent Dose
ICH GCP	International Conference on Harmonisation—Good Clinical Practise
i.p.	Intraperitoneal
MAD	Multiple Ascending Dose
MoA	Mechanism of Aciton
MOAA/S	Modified Observer’s Assessment of Alertness/Sedation
NREM	Non-Rapid Eye Movement
PAM	Positive Allosteric Modulator
PBPK	Physiologically Based Pharmacokinetic
PD	Pharmacodynamic
PK	Pharmacokinetic
qEEG	Quantitative EEG
Rac	Accumulation Ratio
REM	Rapid Eye Movement
SAD	Single Ascending Dose
SAEs	Serious Adverse Events
SD	Sprangue Dawley (Rats)
SEM	Standard Error of the Mean
SPV	Saccadic Peak Velocity
SRT	Saccadic Reaction Time
T_max_	Time to Maximum Concentration
T_1/2_	Elimination Half-life
VAS	Visual Analogue Scale
Vz/F	Apparaent Volume of Distribution
VVLT	Visual Verbal Learning Task
WMO	Dutch Act on Medical Research Involving Human Subjects “Wet medisch-wetenschappelijk onderzoek met mensen”

## Appendix A

### Appendix A.1

The elevated plus maze (EPM) test was conducted at PsychoGenics, Inc. (Paramus, NJ, USA). The maze apparatus (Hamilton Kinder) consisted of two closed arms (15.75 cm h × 10.8 cm w × 50.17 cm l) and two open arms (10.8 cm w × 50.17 cm l), forming a cross, with a square center platform (6 × 6 cm). All visible surfaces were made of black acrylic. Each arm of the maze was placed on a support column 85.09 cm above the floor. Antistatic black vinyl curtains (7′ tall) surrounded the EPM to make a 7′ × 7′ enclosure. Rats were housed in ventilated cages and acclimated for up to 1 week prior to commencing the study. All rats were examined and weighed prior to initiation of this study to assure adequate health and suitability. During the course of this study, 12/12 light/dark cycle was maintained. The room temperature was maintained between 20 and 23 °C with a relative humidity maintained around 50%. Chow and water were provided ad libitum for the duration of this study. All testing was performed during the rat’s light cycle phase. Each rat was randomly assigned across the treatment groups. Rats were acclimated to the experimental room for at least 1 h prior to the test.

### Appendix A.2

Quantitative EEG (qEEG) was conducted at PsychoGenics, Inc. (Paramus, NJ, USA). EEG recordings were obtained using DSI DataQuest ART. Rats were assigned unique identification numbers and housed with 3 rats per cage in polycarbonate cages with micro-isolator filter tops. All rats were examined and weighed prior to initiation of this study to assure adequate health and suitability. During this study, 12/12 light/dark cycles were maintained. The room temperature was maintained between 20 and 23 °C with a relative humidity maintained around 50%. Chow and water were provided ad libitum for the duration of this study. Following surgery, rats were single housed. After a period of recovery (7–10 days), animals were transferred to an EEG recording room and placed over a receiver for recordings. ENX-102 was formulated in 0.5% Tylose MH 300 solution and dosed orally. Lorazepam, the benzodiazepine control, administered i.p. at 1 mg/kg, was dissolved in saline. After four days of washout, recordings were performed on a Monday and Friday dosing/recording schedule. EEG recordings started in the lights-off period and dosing occurred 4 h after lights were turned off. Animals were habituated to dosing (vehicle dosing) for 2 days before any data collection took place.

### Appendix A.3

For sleep scoring, raw EEG recordings were manually scored using Neuroscore software version 3.3 (Data Sciences International) to identify sleep stages: active wake, quiet wake, non-rapid eye movement (NREM), and rapid eye movement (REM). Using NeuroScore, artifacts were removed offline from the data and sleep stages assigned manually for every 10 s epoch using EEG, EMG, and locomotor activity (LMA) by conventional methods as previously described [62,63,64,65] using the fronto-parietal EEG, LMA, and EMG: active wake (less regular, low-amplitude EEG with high EMG and LMAactivity). REM sleep (stable, low-amplitude waves dominated by theta (4–8 Hz) with near absent EMG and no LMA). Sleep stage data were exported from a Neuroscore report template of sleep state time per each 15 min period (2 h pre-dose to 4 h post-dose). The onset of the first sleep and first REM were also reported directly from a Neuroscore report template.

## Appendix B

A serious adverse event (SAE) is any untoward medical occurrence that, at any dose, meets one or more of the following criteria:

- Results in death;
- Is life threatening;
  NOTE: The term “life-threatening” in the definition of “serious” refers to an event in which the subject was at risk of death at the time of the event; it does not refer to an event which hypothetically might have caused death if it were more severe.
- Requires inpatient hospitalization or prolongation of existing hospitalization;
- Results in persistent or significant disability/incapacity;
- Is a congenital anomaly/birth defect;
- Important medical events that may not result in death, be life-threatening, or require hospitalization may be considered serious when, based on appropriate medical judgment, they may jeopardize the subject and may require medical or surgical intervention to prevent one of the outcomes listed in this definition. Examples of such medical events include allergic bronchospasm requiring intensive treatment in an emergency room or at home, blood dyscrasias or convulsions that do not result in subject hospitalization, or the development of study drug dependency or drug abuse.

## Appendix C

### Appendix C.1. Eye Movements

Saccadic peak velocity (SPV) and smooth pursuit eye movements were assessed to evaluate sedation. SPV is known to be highly sensitive to sedation [27] and can indicate anxiolytic properties of benzodiazepines [35]. Smooth pursuit measures neurophysiological function and is sensitive to benzodiazepines [18], the a1-preferring zolpidem [66], and some α2-/3 subunit-selective GABA-A-R modulators [18]. SPV was measured using a computerized system [67]. Volunteers followed a horizontally jumping light source with their eyes. Smooth pursuit eye movements were recorded using the same set up as for the saccadic eye movements [68].

### Appendix C.2. Adaptive Tracking

The adaptive tracking test measures eye-hand coordination and sustained attention and is sensitive to CNS effects of alcohol, psychoactive drugs, and sleep deprivation [26]. In this study, the test followed Borland and Nicholson’s specifications [69]. Participants used a joystick to keep a dot inside a randomly moving circle on a screen; the circle’s speed increased with success and decreased with failure.

### Appendix C.3. Pupillometry

Pupil size was measured to monitor any drug effect, which provided evidence of CNS penetration. Pupillometry was performed as previously described [70]; a simultaneous photo of both eyes measured the pupil/iris diameter ratio (mm).

### Appendix C.4. Body Sway

Body sway measures postural (in)stability and can be impaired by muscle relaxation, which can be mediated by GABA-A-R containing α5 subunits [71] as well as central sedation mediated by α1 activation. At CHDR, the method has been used to demonstrate effects of sleep deprivation [72], alcohol [73], benzodiazepines [35], and other psychoactive agents (data on file or cite Groeneveld et al. 2016 [26]). Body sway was assessed using methods previously described [23] via an apparatus similar to the Wright ataxia meter. A string was affixed to the waist to capture all body movements over a 2-min period, quantifying sway in millimeters on a digital display.

### Appendix C.5. Bond and Lader Visual Analog Scale

The Bond and Lader VAS, originally described by Norris [74], is commonly used to quantify subjective effects of a variety of sedative agents. It measures subjective drug effects in the domains of mood, self-control, and sedation [75]. The VAS alertness is noted as a sensitive parameter for benzodiazepines and is indicative for α1-modulation [35].

### Appendix C.6. Visual Verbal Learning Task

The visual verbal learning task (VVLT) contains three different subtests covering basic aspects of verbal learning and memory—acquisition, consolidation, storage, and retrieval of novel information -and has been previously used to characterize GABA-A PAMs [18]. In total, four versions of the VVLT were used. A training version was used for screening purposes, with separate versions administered on day -1 for baseline measurement, on day 1 at the time of the first administration, and at the final administration on day 12. The VVLT assessments on days 1 and 12 were scheduled so that immediate recall occurred around the Tmax of ENX-102. Participants were visually presented 30 words in three consecutive word trials. Immediately after each trial, participants were invited to orally recall as many words as possible to assess VVLT’s immediate recall. VVLT delayed recall and delayed recognition were assessed 3 and 10 h post-dose. For delayed recall, participants were asked to recall as many words as possible from the list presented during the immediate recall task. Delayed recognition, also known as the memory recognition test, involved visually presenting 30 previously shown words and 30 distractors to evaluate memory retention. Participants were required to identify the words presented during the immediate recall task.

## Appendix D

Resting-state qEEG recordings were performed with participants in two conditions: eyes open and eyes closed. These recordings were conducted in a sequence with other NeuroCart assessments. Each recording session alternated between 64-s periods of eyes open and eyes closed. During this time, participants were instructed to face a featureless wall, refrain from staring, avoid head and eye movements, and suppress eye blinks. This procedure was implemented to minimize extraneous motion artifacts during the recordings.

The qEEG assessments were conducted in eight consecutive blocks of eight periods each, utilizing standard pharmaco-qEEG lead placement, with the same common ground electrode as used in the eye movement registration. Continuous EEG recordings were obtained using a 40-channel recording system (Refa-40, TMSi B.V., The Netherlands). Electrodes were placed according to the international 10–20 system (using a 24-lead cap, TMSi B.V.), with the exception that electrodes at the earlobes (A1 and A2), which were replaced by electrodes at the mastoid sites (M1 and M2). The scalp electrode impedance was maintained below 5 kΩ to ensure good signal quality. The ground electrode was placed at the anterior frontal midline zero (AFz).

To detect ocular artifacts, electrooculography (EOG) was recorded in addition to the EEG. Two Ag/AgCl electrodes were placed at the outer canthi of both eyes, and two additional electrodes were placed approximately 2 cm above and below the right eye to capture vertical and horizontal eye movements.

All signals were sampled at a rate of 1024 Hz and were filtered prior to storage using a first-order recursive high-pass filter with a cut-off frequency of 0.1 Hz to eliminate low-frequency drift. Digital markers were recorded by the amplifier to indicate the start and end of each eye state (eyes open and eyes closed).

EEG recordings were analyzed for power spectral density (PSD) using fast Fourier transform (FFT) analysis. The raw signals were first band-pass filtered using a third-order Butterworth filter, with cutoff frequencies set at 0.1 Hz (low) and 45.0 Hz (high) to remove both high-frequency noise and low-frequency drift. After filtering, the data were segmented into 4-s epochs to allow for time-domain analysis. Epochs containing ocular artifacts (e.g., eye blinks or movements) were identified and excluded to ensure the data used in the subsequent analyses were artifact-free. For the remaining clean epochs, power spectral density (PSD) was calculated for each segment. The PSD values were then averaged across all epochs for each participant under both eyes open and eyes closed conditions. The resulting PSDs were subdivided into the following frequency bands: δ (0.5–6.0 Hz), θ (6.0–8.5 Hz), α (8.5–12.5 Hz), β (12.5–30.0 Hz), and γ (30.0–40.0 Hz). Fast Fourier transform (FFT) analysis was applied to each epoch to calculate the amplitude sum within each frequency band for the electrodes of interest (Fz-Cz, Pz-O1, and Pz-O2).
