# Supplementary material for: Biomarker-Based Pharmacological Characterization of ENX-102, a Novel α2/3/5 Subtype-Selective GABAA Receptor Positive Allo-Steric Modulator: Translational Insights from Rodent and Human Studies
Source: Cells. 2025 Oct 10;14(20):1575. doi: 10.3390/cells14201575 (PMC12564412; doi:10.3390/cells14201575)
Supplement: Supplementary file 1 [file cells-14-01575-s001.zip › cells-3817231-supplementary.pdf]

Supplementary Material

1.1 Supplementary Figure S1

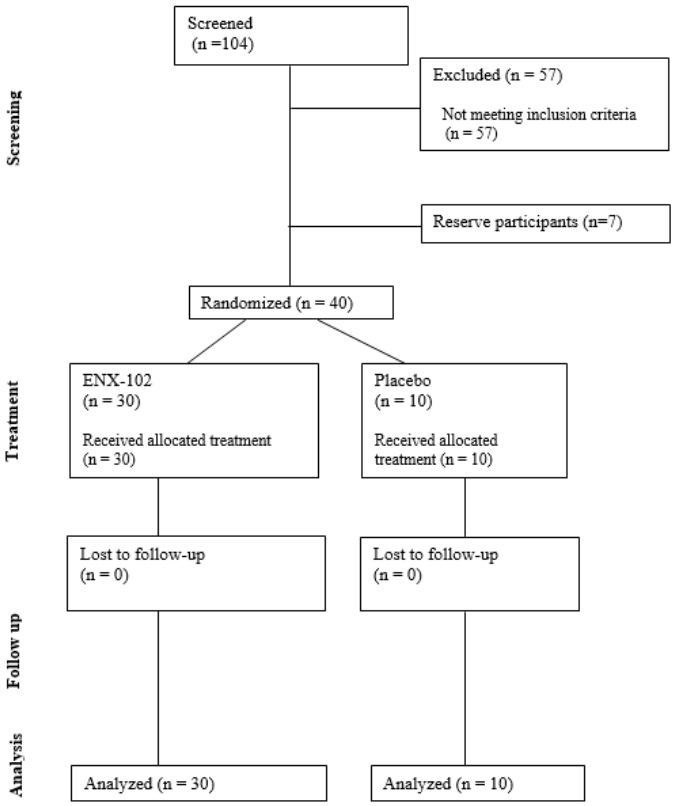

Figure S1: Consort diagram of randomized participants.

1.2 Supplementary Table S1

	ENX-102						
**Characteristic**	**0.5 mg (N = 6)**	**1.0 mg (N = 6)**	**1.5 mg (N = 6)**	**2.0 mg (N = 6)**	**5.0 mg (N = 6)**	**Placebo (N = 10)**	**All Participants (N = 40)**
Sex (Female/Male)	3 (50.0%)/ 3 (50.0%)	3 (50.0%)/ 3 (50.0%)	2 (33.3%)/ 4 (66.7%)	3 (50.0%)/ 3 (50.0%)	2 (33.3%)/ 4 (66.7%)	5 (50.0%)/ 5 (50.0%)	18 (45.0%)/ 22 (55.0%)
Race (Asian/Black or African American/Multiple/Other/White)	1 (16.7%)/ 1 (16.7%)/ 1 (16.7%)/ 1 (16.7%)/ 2 (33.3%)	0/ 0/ 1 (16.7%)/ 0/ 5 (83.3%)	0/ 1(16.7%)/ 0/ 0/ 5 (83.3%)	0/ 0/ 0/ 0/ 6 (100.0%)	1 (16.7%)/ 1 (16.7%)/ 0/ 0/ 4 (66.7%)	0/ 0/ 0/ 1 (10.0%) 9 (90.0%)	2 (5.0%)/ 3 (7.5%)/ 2 (5.0%)/ 2 (5.0%)/ 31 (77.5%)
Age (years) Mean (SD)/ Min, Max	33.2 (7.7)/ 28, 48	32.5 (12.3)/ 19, 51	28.2 (12.3)/ 19, 52	33.8 (6.4)/ 28, 43	28.3 (6.2)/ 19, 36	28.4 (7.4)/ 22, 42	30.5 (8.7)/ 19, 52
Weight (kg) Mean (SD)/ Min, Max	78.1 (18.4)/ 53.7, 105.1	74.8 (10.5)/ 63.6, 91.4	75.2 (15.4)/ 65.0, 106.2	77.7 (7.8)/ 68.2, 90.2	72.2 (15.3)/ 53.2, 94.2	76.0 (15.2)/ 62.5, 104.9	75.7 (13.5)/ 53.2, 106.2
Height (cm) (Mean (SD)/ Min, Max	172.0 (12.8)/ 156.8, 191.8	175.8 (7.5)/ 162.2, 183.2	175.1 (8.9)/ 163.0, 186.1	172.6 (8.8)/ 163.3, 186.4	173.7 (9.4)/ 160.9, 188.0	172.4 (7.9)/ 160.7, 184.2	173.5 (8.7)/ 156.8, 191.8
BMI (kg/m^2) Mean (SD)/ Min, Max	26.5 (6.0)/ 19.8, 34.0	24.5 (5.4)/ 19.9, 34.7	24.5 (4.0)/ 20.1, 31.5	26.3 (4.1)/ 23.1, 33.8	23.8 (3.8)/ 18.5, 29.1	25.6 (4.8)/ 21.2, 34.6	25.2 (4.5)/ 18.5, 34.7

Table S1: Demographic and baseline Characteristics of Study participants by treatment: Values are presented as n (%) for categorical variables and as mean (SD) with minimum and maximum values for continuous variables. ENX-102 was administered at five dose levels (0.5 - 5.0 mg) with N = 6 participants per group; placebo group included N = 10. All participants (N = 40) were healthy adults. Percentages may not total 100% due to rounding. BMI = Body Mass Index; SD = Standard Deviation.

1.3 Supplementary Figure S2

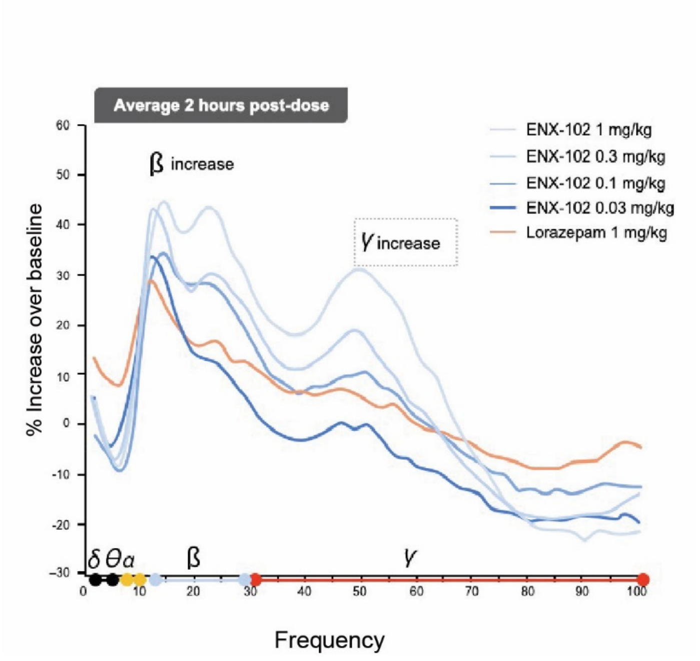

Figure S2. qEEG signature in rats of ENX-102 at 2 hours post-dose

Frequency and magnitude of brain oscillations following treatment with ENX-102 at four dose levels, 0.03 to 1 mg/kg (shades of blue), was measured using quantitative EEG and normalized over baseline. Lorazepam (benzodiazepine) was used as a reference comparator (orange line). ENX-102 produced dose-dependent changes in EEG spectral power changes. ENX-102 increased EEG spectral power in the higher frequency, β and γ bands, while decreasing power in the lower frequency, δ and θ bands.

1.4 Supplementary Figure S3

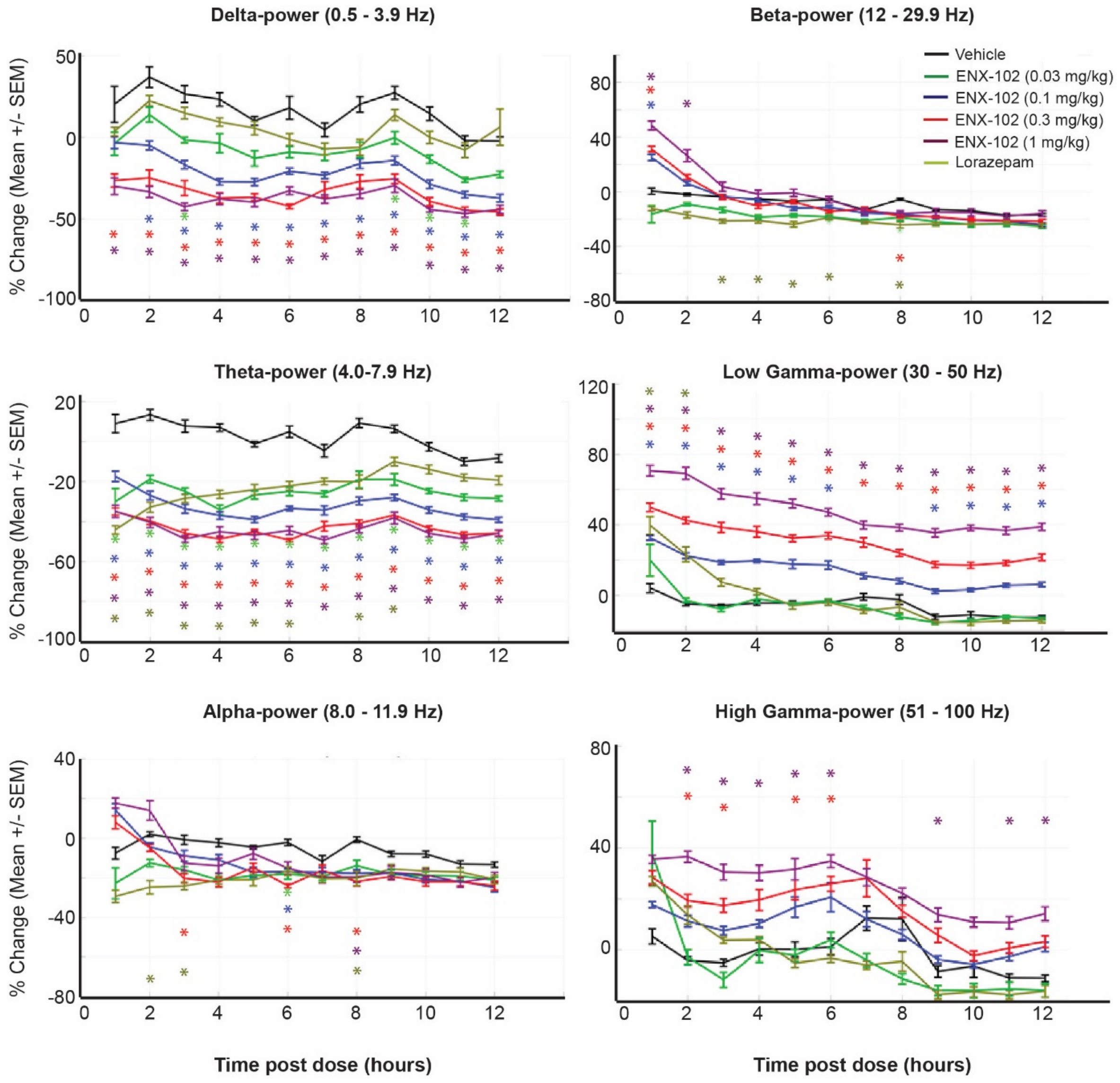

Figure S3: qEEG in rats at non-rapid eye movement sleep (NREM) (0-12 hours post dose): Doses tested: vehicle, ENX-102 (0.03, 0.1, 0.3, 1.0 mg/kg), and lorazepam (1.0 mg/kg). Percent change (mean ± SEM) in δ, θ, α, β, low-γ, high-γ, bands compared to baseline. Statistical significance indicated with * at different timepoints for each frequency band.

1.5 Supplementary Figure S4

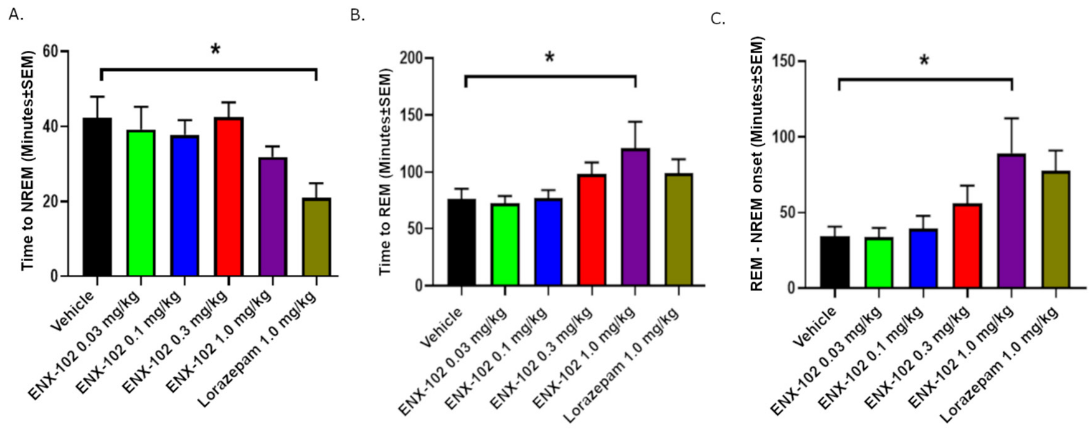

Figure S4: Onset of Rapid Eye Movement (REM), Non-Rapid Eye Movement (NREM), and latency for REM for vehicle, ENX-102 and Lorazepam in rats. Onset is the time (minutes) when the first episode of REM begins. Latency is defined as the time to onset of the first episode of REM following the onset of NREM (i.e. TREM – TNREM = REM Latency). (A) Lorazepam (1.0 mg/kg) significantly decreased the time to NREM compared to the Vehicle group, whereas ENX-102 had no significant effect on the time to NREM at any dose. (B) ENX-102 (1.0 mg/kg) significantly increased the time to REM compared to Vehicle, whereas ENX-102 < 1.0 mg/kg and Lorazepam (1.0 mg/kg) showed no significant changes. (C) ENX-102 (1.0 mg/kg) significantly increased the time to REM compared to vehicle, whereas ENX-102 < 1.0 mg/kg and Lorazepam (1.0 mg/kg) showed no significant changes. *$p < 0.05$ indicates ENX-102 versus vehicle (ANOVA with Dunnett's multiple comparison's test).

1.6 Supplementary Figure S5

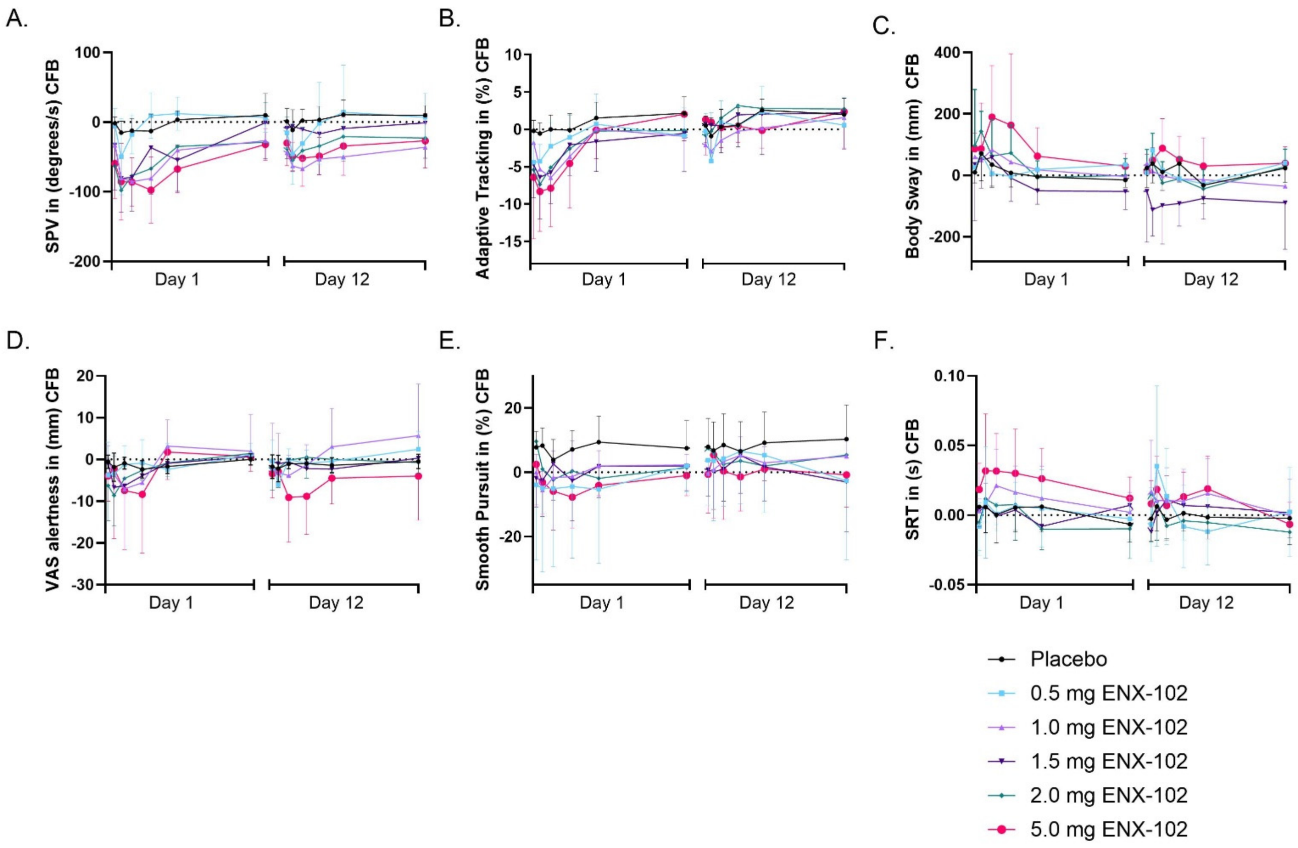

SPV: Saccadic Peak Velocity; s: seconds; SRT: Saccadic Reaction Time; CFB: Change From Baseline; VAS: Visual Analogue Scale; mm: millimeter

Figure S5: Mean (standard Pharmacodynamic parameters of the NeuroCart saccadic peak velocity (SPV), adaptive tracking, smooth pursuit eye movements, body sway, VAS Alertness) measured at 0.5, 1, 1.5, 3, 6, 8, 10, and 24 hours on Day 1 and on Day 12. SPV: statistical significant decrease at day 1 for ENX-102 1.0 mg, 1.5 mg, 2.0 mg, and 5.0 mg ($p < 0.001$); statistical significant decrease at day 12 for ENX-102 1.0 mg ($p<0.001$), 2.0 mg ($p<0.01$), and 5.0 mg ($p < 0.01$); Adaptive Tracking: statistical significant decrease at day 1 for ENX-102 1.0 mg ($p<0.05$), 1.5 mg ($p<0.01$), 2.0 mg ($p<0.01$), and 5.0 mg ($p < 0.001$); no statistical significant decrease at day 12. Body sway: Body sway: No statistically significant change at day 1; statistical significant decrease at day 12 for ENX-102 1.5 mg ($p<0.05$). VAS alertness: significant decrease at day 1 for ENX-102 2.0 mg ($p<0.05$); statistical significant decrease at day 12 for ENX-102 5.0 mg.

1.7 Supplementary Table S2

Dose (mg/kg)	T_{max} (h)	C_{max} (ng/mL)	AUC_{0-t} (ng*h/mL)	$T_{1/2}$ (h)	HED
0.1	4.0	35	*487.5*	N.R.	~1
0.32	1.5	140	2185	15.8	~3
1.0	4	362	*4875*	N.R.	~10
1.4	4.5	602	9550	15.45	~14
3.2	4.5	1505	25850	19.1	~31

Table S2: Pharmacokinetics for ENX-102 in rats *(Data on file.)* Pharmacokinetic parameters for oral doses ranging from 0.1 mg/kg to 3.2 mg/kg include time to maximum concentration (T_{max}) in hours (h), maximum plasma concentration (C_{max}) in ng/mL, and area under the concentration-time curve from time zero to time t (AUC_0–t)—*values extrapolated from the Investigator's Brochure are italicized*. Also reported are the elimination half-life ($T_1/_2$) and the human equivalent dose (HED) calculated for a 60 kg human.

1.8 Supplementary Table S3

	**0.5 mg ENX-102**		**1.0 mg ENX-102**		**1.5 mg ENX-102**		**2.0 mg ENX-102**		**5.0 mg ENX-102**		***Placebo***	
	**# of AE**	**N**	**# of AEs**	**N**	**# of AEs**	**N**	**# of AEs**	**N**	**# of AEs**	**N**	**# of AEs**	**N**
**Total AEs N = 408**												
Day 1	*10*	*6*	*6*	*4*	*17*	*6*	*12*	*6*	*11*	*6*	*3*	*2*
Day 12	*5*	*5*	*6*	*4*	*5*	*5*	*5*	*2*	*3*	*3*	*6*	*3*
Total number of AE during the whole study	*65*	*6*	*73*	*6*	*99*	*6*	*64*	*6*	*53*	*6*	*54*	*10*

Table S3: Summary of the total number of Adverse Events (AEs), and the five most commonly reported AEs per treatment group on Day 1, Day 12 and Days 1 through 12.

1.9 Supplementary Table S4

System Organ Class/ Preferred Term	0.5mg ENX-102 (N=6) Participants N (%)	Events N	1.0mg ENX-102 (N=6) Participants N (%)	Events N	1.5mg ENX-102 (N=6) Participants N (%)	Events N	2.0mg ENX-102 (N=6) Participants N (%)	Events N
ANY EVENTS	6 (100.0%)	65	6 (100.0%)	73	6 (100.0%)	99	6 (100.0%)	64
Cardiac disorders	0	0	0	0	0	0	0	0
Bradycardia	0	0	0	0	0	0	0	0
Eye disorders	0	0	0	0	1 (16.7%)	1	0	0
Eye irritation	0	0	0	0	1 (16.7%)	1	0	0
Low visual acuity	0	0	0	0	0	0	0	0
Gastrointestinal disorders	0	0	1 (16.7%)	1	3 (50.0%)	3	1 (16.7%)	1
Acid reflux (esophageal)	0	0	0	0	0	0	1 (16.7%)	1
Loose stools	0	0	0	0	2 (33.3%)	2	0	0
Nauseous	0	0	0	0	1 (16.7%)	1	0	0
Stomach discomfort	0	0	1 (16.7%)	1	0	0	0	0
General disorders and administration site conditions	6 (100.0%)	13	6 (100.0%)	35	6 (100.0%)	32	5 (83.3%)	19
Fatigue	5 (83.3%)	10	5 (83.3%)	19	6 (100.0%)	28	5 (83.3%)	17
Feeling abnormal	0	0	2 (33.3%)	7	0	0	0	0
Feeling cold	0	0	1 (16.7%)	6	0	0	0	0
Feeling relaxed	2 (33.3%)	2	1 (16.7%)	3	2 (33.3%)	3	1 (16.7%)	2
Hangover (excl alcohol)	1 (16.7%)	1	0	0	0	0	0	0
Shivers	0	0	0	0	1 (16.7%)	1	0	0
Infections and infestations	3 (50.0%)	3	0	0	3 (50.0%)	4	0	0
COVID-19	2 (33.3%)	2	0	0	0	0	0	0
Common cold	0	0	0	0	1 (16.7%)	1	0	0
Gastroenteritis	1 (16.7%)	1	0	0	0	0	0	0
Nasopharyngitis	0	0	0	0	2 (33.3%)	2	0	0
Upper respiratory tract infection	0	0	0	0	1 (16.7%)	1	0	0

System Organ Class/ Preferred Term	5.0mg ENX-102 (N=6)		Placebo (N=10)		All ENX-102 (N=30)	
	Participants N (%)	Events N	Participants N (%)	Events N	Participants N (%)	Events N
ANY EVENTS	6 (100.0%)	53	9 (90.0%)	54	30 (100.0%)	354
Cardiac disorders	1 (16.7%)	1	0	0	1 (3.3%)	1
Bradycardia	1 (16.7%)	1	0	0	1 (3.3%)	1
Eye disorders	0	0	1 (10.0%)	1	1 (3.3%)	1
Eye irritation	0	0	0	0	1 (3.3%)	1
Low visual acuity	0	0	1 (10.0%)	1	0	0
Gastrointestinal disorders	0	0	0	0	5 (16.7%)	5
Acid reflux (esophageal)	0	0	0	0	1 (3.3%)	1
Loose stools	0	0	0	0	2 (6.7%)	2
Nauseous	0	0	0	0	1 (3.3%)	1
Stomach discomfort	0	0	0	0	1 (3.3%)	1
General disorders and administration site conditions	5 (83.3%)	7	4 (40.0%)	16	28 (93.3%)	106
Fatigue	5 (83.3%)	6	4 (40.0%)	15	26 (86.7%)	80
Feeling abnormal	0	0	0	0	2 (6.7%)	7
Feeling cold	0	0	0	0	1 (3.3%)	6
Feeling relaxed	1 (16.7%)	1	1 (10.0%)	1	7 (23.3%)	11
Hangover (excl alcohol)	0	0	0	0	1 (3.3%)	1
Shivers	0	0	0	0	1 (3.3%)	1
Infections and infestations	0	0	2 (20.0%)	2	6 (20.0%)	7
COVID-19	0	0	1 (10.0%)	1	2 (6.7%)	2
Common cold	0	0	0	0	1 (3.3%)	1
Gastroenteritis	0	0	1 (10.0%)	1	1 (3.3%)	1
Nasopharyngitis	0	0	0	0	2 (6.7%)	2
Upper respiratory tract infection	0	0	0	0	1 (3.3%)	1

System Organ Class/ Preferred Term	0.5mg ENX-102 (N=6)		1.0mg ENX-102 (N=6)		1.5mg ENX-102 (N=6)		2.0mg ENX-102 (N=6)	
	Participants N (%)	Events N	Participants N (%)	Events N	Participants N (%)	Events N	Participants N (%)	Events N
Injury, poisoning and procedural complications	1 (16.7%)	1	0	0	1 (16.7%)	1	1 (16.7%)	1

Bruising of leg	0	0	0	0	0	0	0	0
Other and unspecified open wound of head without mention of complication	1 (16.7%)	1	0	0	0	0	0	0
Post procedural hematoma	0	0	0	0	0	0	1 (16.7%)	1
Procedural site pain	0	0	0	0	0	0	0	0
Skin wound	0	0	0	0	1 (16.7%)	1	0	0
Investigations	1 (16.7%)	1	0	0	0	0	1 (16.7%)	1
Blood creatine phosphokinase increased	0	0	0	0	0	0	0	0
CPK increase	0	0	0	0	0	0	1 (16.7%)	1
Elevated liver enzymes	1 (16.7%)	1	0	0	0	0	0	0
Musculoskeletal and connective tissue disorders	1 (16.7%)	1	1 (16.7%)	1	0	0	0	0
Back stiffness	0	0	0	0	0	0	0	0
Muscle pain	0	0	0	0	0	0	0	0
Muscle weakness	0	0	0	0	0	0	0	0
Neck pain	1 (16.7%)	1	0	0	0	0	0	0
Shoulder pain	0	0	1 (16.7%)	1	0	0	0	0

	**5.0mg ENX-102 (N=6)**		**Placebo (N=10)**		**All ENX-102 (N=30)**	
**System Organ Class/ Preferred Term**	**Participants N (%)**	**Events N**	**Participants N (%)**	**Events N**	**Participants N (%)**	**Events N**
Injury, poisoning and procedural complications	2 (33.3%)	2	1 (10.0%)	1	5 (16.7%)	5
Bruising of leg	1 (16.7%)	1	0	0	1 (3.3%)	1
Other and unspecified open wound of head without mention of complication	0	0	0	0	1 (3.3%)	1
Post procedural hematoma	0	0	0	0	1 (3.3%)	1
Procedural site pain	1 (16.7%)	1	1 (10.0%)	1	1 (3.3%)	1
Skin wound	0	0	0	0	1 (3.3%)	1
Investigations	0	0	1 (10.0%)	1	2 (6.7%)	2
Blood creatine phosphokinase increased	0	0	1 (10.0%)	1	0	0
CPK increase	0	0	0	0	1 (3.3%)	1
Elevated liver enzymes	0	0	0	0	1 (3.3%)	1
Musculoskeletal and connective tissue disorders	1 (16.7%)	2	2 (20.0%)	2	3 (10.0%)	4
Back stiffness	0	0	1 (10.0%)	1	0	0
Muscle pain	1 (16.7%)	2	0	0	1 (3.3%)	2
Muscle weakness	0	0	1 (10.0%)	1	0	0
Neck pain	0	0	0	0	1 (3.3%)	1
Shoulder pain	0	0	0	0	1 (3.3%)	1

System Organ Class/ Preferred Term	0.5mg ENX-102 (N=6)		1.0mg ENX-102 (N=6)		1.5mg ENX-102 (N=6)		2.0mg ENX-102 (N=6)	
	**Participants N (%)**	**Events N**	**Participants N (%)**	**Events N**	**Participants N (%)**	**Events N**	**Participants N (%)**	**Events N**
Nervous system disorders	6 (100.0%)	35	6 (100.0%)	26	6 (100.0%)	45	6 (100.0%)	36
Balance disorder	0	0	0	0	3 (50.0%)	5	1 (16.7%)	1
Bradykinesia	0	0	0	0	1 (16.7%)	2	0	0
Concentration impairment	2 (33.3%)	3	2 (33.3%)	3	3 (50.0%)	5	0	0
Dizziness	1 (16.7%)	3	0	0	1 (16.7%)	3	1 (16.7%)	3
Dizziness postural	2 (33.3%)	5	2 (33.3%)	3	2 (33.3%)	5	1 (16.7%)	3
Forgetfulness	0	0	0	0	0	0	1 (16.7%)	1
Headache	0	0	4 (66.7%)	6	1 (16.7%)	2	2 (33.3%)	2
Lightheadedness	0	0	0	0	0	0	0	0
Sleep paralysis	0	0	0	0	0	0	1 (16.7%)	1
Somnolence	6 (100.0%)	23	5 (83.3%)	14	5 (83.3%)	23	6 (100.0%)	25
Syncope	1 (16.7%)	1	0	0	0	0	0	0
Psychiatric disorders	4 (66.7%)	7	3 (50.0%)	4	2 (33.3%)	6	2 (33.3%)	2
Anxiety	1 (16.7%)	1	0	0	0	0	0	0
Blunted affect	0	0	0	0	0	0	0	0
Bradyphrenia	0	0	0	0	1 (16.7%)	2	1 (16.7%)	1
Emotional lability	0	0	1 (16.7%)	1	0	0	0	0
Euphoric mood	1 (16.7%)	2	0	0	1 (16.7%)	2	0	0
Feeling down	0	0	1 (16.7%)	1	0	0	0	0
Hypervigilance	1 (16.7%)	1	0	0	0	0	0	0
Insomnia	0	0	0	0	1 (16.7%)	1	0	0
Lack of motivation	1 (16.7%)	1	0	0	0	0	0	0
Nightmare	1 (16.7%)	1	0	0	0	0	0	0
Poor quality sleep	1 (16.7%)	1	2 (33.3%)	2	0	0	0	0
Staring	0	0	0	0	1 (16.7%)	1	0	0
Vivid dreams	0	0	0	0	0	0	1 (16.7%)	1

System Organ Class/ Preferred Term	5.0mg ENX-102 (N=6) Participants N (%)	Events N	Placebo (N=10) Participants N (%)	Events N	All ENX-102 (N=30) Participants N (%)	Events N
Nervous system disorders	6 (100.0%)	32	6 (60.0%)	25	30 (100.0%)	174
Balance disorder	1 (16.7%)	2	0	0	5 (16.7%)	8
Bradykinesia	0	0	0	0	1 (3.3%)	2
Concentration impairment	0	0	0	0	7 (23.3%)	11
Dizziness	3 (50.0%)	8	0	0	6 (20.0%)	17
Dizziness postural	2 (33.3%)	3	1 (10.0%)	14	9 (30.0%)	19
Forgetfulness	0	0	0	0	1 (3.3%)	1
Headache	1 (16.7%)	1	2 (20.0%)	4	8 (26.7%)	11
Lightheadedness	1 (16.7%)	2	0	0	1 (3.3%)	2
Sleep paralysis	0	0	0	0	1 (3.3%)	1
Somnolence	5 (83.3%)	16	5 (50.0%)	7	27 (90.0%)	101
Syncope	0	0	0	0	1 (3.3%)	1
Psychiatric disorders	2 (33.3%)	2	1 (10.0%)	2	13 (43.3%)	21
Anxiety	0	0	0	0	1 (3.3%)	1
Blunted affect	0	0	1 (10.0%)	1	0	0
Bradyphrenia	0	0	0	0	2 (6.7%)	3
Emotional lability	1 (16.7%)	1	0	0	2 (6.7%)	2
Euphoric mood	0	0	0	0	2 (6.7%)	4
Feeling down	0	0	0	0	1 (3.3%)	1
Hypervigilance	1 (16.7%)	1	1 (10.0%)	1	2 (6.7%)	2
Insomnia	0	0	0	0	1 (3.3%)	1
Lack of motivation	0	0	0	0	1 (3.3%)	1
Nightmare	0	0	0	0	1 (3.3%)	1
Poor quality sleep	0	0	0	0	3 (10.0%)	3
Staring	0	0	0	0	1 (3.3%)	1
Vivid dreams	0	0	0	0	1 (3.3%)	1

System Organ Class/ Preferred Term	0.5mg ENX-102 (N=6) Participants N (%)	Events N	1.0mg ENX-102 (N=6) Participants N (%)	Events N	1.5mg ENX-102 (N=6) Participants N (%)	Events N	2.0mg ENX-102 (N=6) Participants N (%)	Events N
Respiratory, thoracic and mediastinal disorders	1 (16.7%)	2	1 (16.7%)	2	3 (50.0%)	4	0	0
Bleeding nose	0	0	1 (16.7%)	2	0	0	0	0

Nasal congestion	0	0	0	0	1 (16.7%)	1	0	0
Nosebleed	0	0	0	0	1 (16.7%)	1	0	0
Yawning	1 (16.7%)	2	0	0	1 (16.7%)	2	0	0
Skin and subcutaneous tissue disorders	1 (16.7%)	1	3 (50.0%)	3	1 (16.7%)	1	0	0
Dry skin	0	0	2 (33.3%)	2	1 (16.7%)	1	0	0
Erythema	1 (16.7%)	1	0	0	0	0	0	0
Erythematous skin rash	0	0	1 (16.7%)	1	0	0	0	0
Itch	0	0	0	0	0	0	0	0
Vascular disorders	1 (16.7%)	1	1 (16.7%)	1	1 (16.7%)	2	3 (50.0%)	4
Hematoma	0	0	0	0	0	0	0	0
Hypotension asymptomatic	0	0	0	0	0	0	0	0
Hypotension orthostatic asymptomatic	1 (16.7%)	1	1 (16.7%)	1	1 (16.7%)	2	3 (50.0%)	3
Hypotension orthostatic symptomatic	0	0	0	0	0	0	0	0
Orthostatic hypotension	0	0	0	0	0	0	1 (16.7%)	1

	**5.0mg ENX-102 (N=6)**		**Placebo (N=10)**		**All ENX-102 (N=30)**	
**System Organ Class/ Preferred Term**	**Participants N (%)**	**Events N**	**Participants N (%)**	**Events N**	**Participants N (%)**	**Events N**
Respiratory, thoracic and mediastinal disorders	0	0	0	0	5 (16.7%)	8
Bleeding nose	0	0	0	0	1 (3.3%)	2
Nasal congestion	0	0	0	0	1 (3.3%)	1
Nosebleed	0	0	0	0	1 (3.3%)	1
Yawning	0	0	0	0	2 (6.7%)	4
Skin and subcutaneous tissue disorders	0	0	1 (10.0%)	1	5 (16.7%)	5
Dry skin	0	0	0	0	3 (10.0%)	3
Erythema	0	0	0	0	1 (3.3%)	1
Erythematous skin rash	0	0	0	0	1 (3.3%)	1
Itch	0	0	1 (10.0%)	1	0	0
Vascular disorders	3 (50.0%)	7	1 (10.0%)	3	9 (30.0%)	15
Hematoma	1 (16.7%)	1	0	0	1 (3.3%)	1
Hypotension asymptomatic	1 (16.7%)	1	0	0	1 (3.3%)	1
Hypotension orthostatic asymptomatic	3 (50.0%)	4	1 (10.0%)	1	9 (30.0%)	11
Hypotension orthostatic symptomatic	0	0	1 (10.0%)	2	0	0
Orthostatic hypotension	1 (16.7%)	1	0	0	2 (6.7%)	2

Table S4: **Summary of Number of subjects with treatment emergent Adverse Events (AEs) and number of treatment emergent AEs by System Organ Class and Preferred Term.**

1.10 Supplementary Table S5

	ENX-102 Dose				
	0.5 mg q.d. N=6 (SD[b])	1.0 mg q.d. N=6 (SD[b])	1.5 mg q.d. N=6 (SD[b])	2.0 mg q.d. N=6 (SD[b])	5.0 mg q.d. N=6 (SD[b])
**DAY 1**					
T_{max} (h)	3.00	3.00	3.00	3.00	4.50
C_{max} (ng/mL)	4.615 (1.6207)	8.497 (2.6517)	18.833 (6.9058)	19.633 (3.9581)	49.700 (13.0807)
CV (%) of C_{max}	35.1	31.2	36.7	20.2	26.3
AUC_{0-24} (ng*h/mL)	54.03 (6.380)	117.81 (27.318)	216.99 (48.171)	243.73 (48.247)	641.92 (125.189)
CV (%) of AUC_{0-24}	11.8	23.2	22.2	19.8	19.5
**DAY 12**					
T_{max} (h)	3.00	3.00	3.00	3.00	3.00
C_{max} (ng/mL)	11.66 (4.2484)	23.333 (5.4213)	41.700 (4.8990)	49.817 (17.2081)	99.120 (13.4591)
CV (%) [e] of C_{max}	36.4	23.2	11.7	34.5	13.6
AUC_{tau} (ng*h/mL)	197.54 (50.534)	410.07 (114.752)	705.65 (84.204)	873.17 (331.256)	1803.45 (242.257)
CV (%) of AUC_{tau}	25.6	28.0	11.9	37.9	13.4
$t_{1/2}$ (h)	65.61 (23.6782)	60.876 (29.2396)	50.526 (17.7754)	61.167 (26.4394)	38.695 (8.7849)
CV (%) of $T_{1/2}$	36.1	48.0	35.2	43.2	22.7
R_{ac}	3.957 (0.5735)	3.542 (0.8647)	3.242 (0.9248)	3.618 (1.4481)	3.019 (0.2961)

AUC0-24 (ng·h/mL): mean area under the plasma concentration-time curve over the last 24-hour dosing interval in ng·h/mL; AUCtau (ng·h/mL): area under the curve from time 0 to steady state in ng·h/mL; Cmax (ng/mL): mean maximum plasma concentration in ng/mL; CV (%): coefficient of variation; q.d. (quaque die): every day; Rac: accumulation ratio for the area under plasma concentration-time curve over the last 24-hour dosing interval; SD: standard deviation; T1/2: half-life time in hours; Tmax (h): median time taken to reach the maximum concentration in hours.

Table S5: Pharmacokinetic parameters of ENX-102 0.5 mg, 1.0 mg, 1.5 mg, 2.0 mg, and 5.0 mg at Day 1 and Day 12.

1.11 Supplementary Table S6

	Dose (mg)														
	0.5			1.0			1.5			2.0			5.0		
	LSM	*Estimated difference from placebo (mm) 95% CI*	*p-value*	LSM	*Estimated difference from placebo (mm) 95% CI*	*p-value*	LSM	*Estimated difference from placebo (mm) 95% CI*	*p-value*	LSM	*Estimated difference from placebo (mm) 95% CI*	*p-value*	LSM	*Estimated difference from placebo (mm) 95% CI*	*p-value*
SPV *(degrees/second)*															
Day 1	445.85	-7.01 (-35.90; 21.88)	0.627	**401.41**	**-51.46 (-80.31; -22.60)**	**<0.001**	**400.46**	**-52.40 (-80.83; -23.97)**	**<0.001**	**387.35**	**-65.51 (-94.43;-36.59)**	**<0.001**	**376.65**	**-76.22 (-106.35;-46.09)**	**<0.001**
Day 12	439.87	-21.80 (-50.62-7.02)	0.134	**410.08**	**-51.59 (-80.44;-22.73)**	**<0.001**	447.17	-14.50 (-42.93;13.93)	0.309	**417.56**	**- 44.11 (-73.03;-15.19)**	**0.004**	**410.28**	**-51.39 (-81.13;-21.65)**	**0.001**
SRT *(seconds)*															
Day 1	0.186	0.0003 (-0.0162;0.0168)	0.9711	0.1939	0.0080 (-0.0082;0.0242)	0.3271	0.1870	0.0011 (-0.0163;0.0185)	0.8987	0.1856	-0.0003 (-0.0168;0.0161)	0.9687	**0.2057**	**0.0198 (0.0032;0.0365)**	**0.0206**
Day 12	0.188	0.007 (-0.01;0.0229)	0.4322	0.1945	0.0133 (-0.0029; 0.0295)	0.1058	0.1889	0.0077 (-0.0097;0.0251)	0.3777	0.1850	0.0038 (-0.0127;0.0202)	0.6476	0.1931	0.0119 (-0.0044;0.0281)	0.1498
Smooth Pursuit *(%)*															
Day 1	**42.51**	**-11.73 (-20.19; -3.27)**	**0.0078**	48.96	-5.27 (-13.9; 3.35)	0.2240	**44.86**	**-9.37 (-17.87; -0.88)**	**0.0314**	48.02	-6.21 (-14.67; 2.25)	0.1456	**44.58**	**-9.66 (-18.14; -1.18)**	**0.0266**
Day 12	51.91	-2.81 (-11.27; 5.65)	0.5059	52.69	-2.04 (-10.66; 6.59)	0.6359	47.16	-7.56 (-16.06; 0.93)	0.0795	50.94	-3.79 (-12.25; 4.67)	0.3711	49.23	-5.50 (-13.98; 2.99)	0.1979
Adaptive Tracking *(%)*															
Day 1	27.590	- 2.420 (-5.271;0.430)	0.0940	**26.478**	**- 3.532 (-6.382;-0.682)**	**0.0164**	**25.541**	**- 4.470 (-7.483;-1.456)**	**0.0046**	**25.611**	**- 4.399 (-7.310;-1.489)**	**0.0040**	**24.350**	**- 5.660 (-8.530;-2.791)**	**< 0.001**
Day 12	29.713	- 0.793 (-3.644;2.058)	0.5776	28.608	- 1.898 (-4.748;0.952)	0.1861	30.825	0.320 (-2.694;3.333)	0.8315	31.043	0.537 (-2.373;3.448)	0.7113	30.367	- 0.139 (-3.009;2.730)	0.9226
Body Sway Logarithm (%)															
Day 1	234.12	8.3 (16.8;41.0)	0.5442	252.61	16.9 (-10.3;52.4)	0.2424	224.49	3.9 (-20.8;36.3)	0.7794	265.64	22.9 (-5.6; 60.1)	0.1225	**312.86**	**44.8 (10.9;89.0)**	**0.0077**
Day 12	216.69	-1.2 (-24.1;28.7)	0.9288	198.33	-9.5 (-30.6;17.9)	0.4503	**153.04**	**-30.2 (-46.8;-8.4)**	**0.0107**	198.67	-9.4 (-30.4;18.0)	0.4558	248.08	13.1 (-13.4;47.8)	0.3563
Pupillometry (pupil: iris ratio)<br>Left *(mm)*															
Day 1	0.48483	-0.00558 (-0.06173;0.05058)	0.8427	**0.43236**	**-0.05805 (-0.11302;-0.00308)**	**0.0389**	**0.43417**	**-0.05623 (-0.011163;-0.00084)**	**0.0468**	0.44764	-0.04277 (-0.09766;0.01213)	0.1239	**0.41766**	**-0.07275 (-0.12781;-0.01769)**	**0.0107**
Day 12	0.49943	0.01285 (-0.04356;0.06925)	0.6492	0.46550	-0.02109 (-0.07606;0.03388)	0.4446	0.53359	0.04701 (-0.00839;0.10240)	0.0945	0.47943	-0.00715 (-0.06205;0.04774)	0.7946	0.50012	0.01353 (-0.04153;0.06860)	0.6236
Pupillometry (pupil: iris ratio)<br>*Right (mm)*															
Day 1	0.47805	-0.02072 (-0.07602; 0.03457)	0.4552	0.45086	-0.04792 (-0.10295; 0.00711)	0.0865	**0.43953**	**-0.05924 (-0.11448; -0.0040)**	**0.0361**	0.45127	-0.04751 (-0.10253; 0.00751)	0.0890	**0.41930**	**-0.07948 (-0.13461; -0.02434)**	**0.0056**
Day 12	0.49037	-0.00104 (-0.05658; 0.05449)	0.9701	0.49742	0.00601 (-0.04903; 0.06104)	0.8274	0.53056	0.03915 (-0.01609; 0.09438)	0.1609	0.49321	0.00180 (-0.05321; 0.05682)	0.9478	0.50696	0.01555 (-0.03959; 0.07069)	0.5737
VAS Alertness (mm)															
Day 1	48.4	-1.0 (-4.3;2,3	0.5554	48.0	-1.5 (-4.8;1.9)	0.3854	47.5	-1.9 (-15.2;1.5	0.2706	**46.1**	**-3.3 (-6.7;-0.0)**	**0.0489**	46.9	-2.5 (-5.8;0.8)	0.1368
Day 12	49.1	-0.2 (-3.5;3.1)	0.8876	49.1	-0.3 (-3.6;3.0)	0.8727	50.0	0.7 (-2.7;4.0)	0.6899	49.8	0.4 (-2.9; 3.7)	0.8028	**45.2**	**-4.2 (-7.5;-0.9)**	**0.0141**
VAS Calmness (mm)															

Day 1	54.4	2.3 (-2.6; 7.2)	0.3398	56.5	4.4 (-0.4; 9.3)	0.0732	55.9	3.8 (-1.2; 8.9)	0.1323	56.2	4.1 (-0.8; 9.0)	0.0974	56.9	4.8 (-0.1; 9.8)	0.0559
Day 12	55.6	4.2 (-0.7; 9.1)	0.0920	54.6	3.2 (-1.7; 8.0)	0.1978	52.6	1.2 (-3.9; 6.2)	0.6446	53.3	1.9 (-3.0; 6.9)	0.4279	53.5	2.1 (-2.9; 7.0)	0.3979
VAS Mood (mm)															
Day 1	50.9	-0.7 (-4.9; 3.6)	0.7543	53.6	2.0 (-2.2; 6.3)	0.3364	52.9	1.3 (-3.1; 5.7)	0.5384	51.3	-0.3 (-4.6; 3.9)	0.8797	52.9	1.3 (-2.9; 5.6)	0.5302
Day 12	52.3	1.2 (-3.0; 5.5)	0.5623	53.7	2.6 (-1.6; 6.9)	0.2173	52.1	2.1 (-2.3; 6.5)	0.3443	52.7	1.7 (-2.6; 5.9)	0.4290	51.9	0.8 (-3.4; 5.1)	0.6927
VVLT Immediate recall correct															
Day 1	6.7	-1.8 (-4.6;0.9)	0.191	8.2	-0.4 (-3,2;2,4)	0.7799	6.5	-2.1 (-4.9;0.7)	0.1424	8.4	-0.2 (-2.9;2.6)	0.9103	**4.7**	**-3.8 (-6.8;-0.9)**	**0.0119**
Day 12	8.7	-1.6 (-4.4; 1.1)	0.2426	10.7	0.3 (-2.5;3.1)	0.8269	9.0	-1.4 (-4.2;1.4)	0.3286	9.2	-1.1 (-3.9;1.7)	0.4221	9.1	-1.2 (-4.1;1.6)	0.3789
VVLT Delayed Word recall correct															
Day 1	9.0	-0.8 (-4.8;3.1)	0.6705	6.5	-3.3 (-7.3;0.6)	0.0943	6.5	-3.3 (-7.2;0.7)	0.1001	7.4	-2.4 (-6.4;1.5)	0.2158	**4.9**	**-4.9 (-9.0;-0.8)**	**0.0189**
Day 12	14.1	0.1 (-3.8; 4.1)	0.9464	11.9	-2.1 (-6.0; 1.8)	0.2838	12.0	-2.0 (-6.0; 1.8)	0.3171	10.3	3.7 (-7.7;0.2)	0.0619	13.5	-0.5 (-4.5;3.4)	0.7824
VVLT Delayed Word Recognition Correct															
Day 1	22.8	-1.6 (-4.5;1.2)	0.2487	24.8	0.3 (-2.5;3.1)	0.8176	21.9	-2.5 (-5.2;0.3)	0.0793	22.4	-2.1 (-5.0; 0.8)	0.1573	**21.4**	**-3.0 (-6.0;-0.0)**	**0.0485**
Day 12	25.9	-0.7 (-3.5; 2.1)	0.6158	25.9	-0.7 (-3.5; 2.1)	0.6384	25.5	-1.1 (-3.9; 1.7)	0.4466	**23.3**	**-3.3 (-6.2; -0.4)**	**0.0265**	23.8	-2.7 (-5.6;-0.1)	0.0622

CI: Confidence Interval; LSM: Least Squares Mean; mg: milligram; SPV: Saccadic Peak Velocity; SRT: Saccadic Reaction Time; VAS: Visual Analogue Scale; VVLT: Visual Verbal Learning Task;

Table S6: Central Nervous System Effects of ENX-102 (0.5 mg, 1.0 mg, 1.5 mg, 2.0 mg, and 5.0 mg) compared to Placebo on Day 1 and Day 12. SPV, SRT, Adaptive Tracking, Body Sway, Pupillometry, VAS Alertness, VAS Calmness, VAS Mood, and VVLT. [1]due to the log transformation, differences measured in millimeters are expressed as percentage change.

1.12 Supplementary Table S7

Eyes open Day 1 (single dose)						
		ENX-102 0.5 mg	ENX-102 1.0 mg	ENX-102 1.5 mg	ENX-102 2.0 mg	ENX-102 5.0 mg
Delta-power (0.5 < 6.0 Hz)	Fz-Cz	32.7 (CI -8.2; 91.9)	3.8 (CI -29.8; 53.4)	-1.4 (CI -31.4; 41.6)	22.5 (CI -16.3; 79.3)	-6.2 (CI -34.5; 34.3)
	Pz-O1	12.0 (CI -15.6; 48.6)	-10.2 (CI -34.0; 22.3)	4.6 (CI -21.1; 38.6)	-12.6 (CI -33.9; 15.7)	23.0 (CI -7.2; 63.1)
	Pz-O2	3.8 (CI-22.7; 39.5)	-8.7 (CI -33.8; 26.0)	-8.2 (CI-32.0; 23.9)	-11.1 (CI -33.7; 19.0)	3.5 (CI -23.0; 39.2)
Theta-power (6.0 < 8.5 Hz)	Fz-Cz	7.6 (CI -21.7; 47.9)	-24.0 (CI -45.9; 6.8)	-25.1 (CI -45.3; 2.4)	24.8 (CI -9.4; 71.9)	-21.3 (CI -42.6; 7.8)
	Pz-O1	-3.4 (CI -33.0; 39.2)	-13.7 (CI -42.1; 28.6)	-20.9 (CI-45.1; 14.1)	-0.6 (CI -31.2; 43.5)	-15.1 (CI-41.2; 22.5)
	Pz-O2	-8.6 (CI -35.8; 30.0)	-0.9 (CI -32.3; 45.2)	-26.9 (CI -48.6; 4.1)	2.7 (CI -28.0; 46.4)	-13.9 (CI -39.5; 22.5)
Alpha-power (8.5 < 12.5 Hz)	Fz-Cz	6.2 (CI -29.7; 60.6)	-10.3 (CI -42.3; 39.6)	**-35.0 (CI -56.9; -2.0; p = 0.0404)***	6.4 (CI -29.7; 60.8)	-13.4 (CI -42.8; 31.0)
	Pz-O1	12.4 (CI -29.3; 78.7)	-12.2 (CI -47.2; 45.9)	-28.5 (CI -55.4; 14.7)	-3.7 (CI -39.6; 53.4	-32.7 (CI -57.8; 7.3)
	Pz-O2	3.0 (CI -32.2; 56.7)	1.5 (CI -35.6; 59.9)	-27.5 (CI -52.6; 10.9)	-1.8 (CI -35.5; 49.5)	-31.7 (CI -55.2; 4.1)
Beta-power (12.5 < 30.0 Hz)	Fz-Cz	29.0 (CI -4.5; 74.2)	11.5 (CI -19.4; 54.1)	12.7 (CI -16.2; 51.7)	**91.3 (CI 42.2; 157.4; p < 0.0001)***	**84.7 (CI 37.1; 148.9; p = 0.0002)***
	Pz-O1	-1.2 (CI -23.4; 27.3)	12.2 (CI -13.7; 45.9)	-7.4 (CI -27.7; 18.6)	18.7 (CI -7.0; 51.6)	3.9 (CI -18.6; 32.6)
	Pz-O2	-4.5 (CI -29.8; 30.1)	16.1 (CI -16.0; 60.6)	-8.4 (CI -32.6; 24.7)	27.6 (CI -5.6; 72.5)	9.9 (CI -18.8; 48.6)
Gamma-power (30.0 < 40.0 Hz)	Fz-Cz	29.4 (CI -6.2; 78.4)	2.5 (CI-26.5; 43.1)	26.5 (CI -6.5; 71.1)	**65.9 (CI 22.6; 124.5; p = 0.0016)***	27.4 (CI-5.9; 72.5)
	Pz-O1	12.8 (CI -22.0; 63.1)	-1.5 (CI -32.0; 42.6)	**-38.3 (CI -56.6; -12.3; p =0.0080)***	**-34.5 (CI -53.8; -7.0; p = 0.0189)***	**-34.6 (CI -54.0; -7.1; p = 0.0185)***
	Pz-O2	0.2 (CI -39.7; 66.6)	-3.9 (CI-42.8; 61.4)	-17.8 (CI -49.7; 34.5)	-16.9 (CI-49.0; 35.3)	-25.6 (CI-54.4; 21.4)
**Eyes closed Day 1 (single dose)**						
		**ENX-102 0.5 mg**	**ENX-102 1.0 mg**	**ENX-102 1.5 mg**	**ENX-102 2.0 mg**	**ENX-102 5.0 mg**
Delta-power (0.5 < 6.0 Hz)	Fz-Cz	-6.1 (CI -31.9; 29.4)	-15.1 (CI -39.2; 18.6)	-21.9 (CI -43.5; 7.9)	4.3 (CI -24.7; 44.5)	-23.0 (CI -44.4; 6.6)
	Pz-O1	-12.5 (CI -35.5; 18.6)	-9.5 (CI -34.5; 25.0)	-14.8 (CI -37.2; 15.5)	-11.7 (CI -34.7; 19.4)	-15.0 (CI -37.4; 15.4)
	Pz-O2	-15.6 (CI -37.8; 14.4)	-6.4 (CI -31.6; 28.0)	-8.9 (CI -32.9; 23.7)	-4.0 (CI -29.1; 29.9)	-8.1 (CI -32.2; 24.6)
Theta-power (6.0 < 8.5 Hz)	Fz-Cz	-8.5 (CI -36.1; 31.2)	-25.4 (CI-48.4; 7.8)	-29.4 (CI-50.8; 1.3)	1.2 (CI -29.5; 45.2)	-27.9 (CI -49.7; 3.4)
	Pz-O1	-14.4 (CI -40.4; 23.0)	-1.2 (CI -31.9; 43.3)	-19.5 (CI -43.9; 15.5)	-17.5 (CI -42.5; 18.5)	-30.4 (CI -51.5; 0.0)
	Pz-O2	-13.0 (CI -39.6; 25.5)	7.9 (CI -25.6; 56.4)	-19.2 (CI -43.9; 16.6)	-16.7 (CI -42.2; 20.0)	-24.1 (CI -47.4; 9.4)
Alpha-power (8.5 < 12.5 Hz)	Fz-Cz	-4.9 (CI -42.1; 56.0)	-22.3 (CI -53.2; 28.8)	-36.7 (CI -61.6; 4.2)	-16.1(CI -48.9; 37.7)	-8.3 (CI -44.3; 51.0)
	Pz-O1	-25.1 (CI -49.5; 11.1)	-3.2 (CI -35.2; 44.8)	**-36.0 (CI -57.1; -4.7; p = 0.0290)***	-26.4 (CI -50.6; 9.6)	-32.4 (CI -54.5; 0.4)
	Pz-O2	-17.2 (CI -43.0; 20.1)	17.4 (CI -19.4; 71.2)	-30.9 (CI -52.6; 0.8)	-22.5 (CI -46.7; 12.8)	-23.3 (CI -47.2; 11.4)
Beta-power (12.5 < 30.0 Hz)	Fz-Cz	29.5 (CI -4.3; 75.3)	18.4 (CI -12.7; 60.6)	18.4 (CI -12.3; 60.0)	**74.9 (CI 29.5; 136.1; p = 0.0005)***	**71.3 (CI 26.9; 131.4; p = 0.0008)***
	Pz-O1	-10.1 (CI -29.4; 14.5)	5.8 (CI -16.9; 34.8)	-16.7 (CI -34.4; 5.7)	8.2 (CI -14.7; 37.4)	5.1 (CI -17.2; 33.4)
	Pz-O2	-6.9 (CI -27.6; 19.7)	18.4 (CI -8.1; 52.5)	-8.8 (CI -29.0; 17.2)	21.6 (CI -5.3; 56.0)	16.0 (CI -9.6; 48.9)
Gamma-power (30.0 < 40.0 Hz)	Fz-Cz	34.6 (CI -0.4; 82.0)	3.1 (CI -23.4; 38.6)	26.9 (CI -4.9; 69.3)	**60.9 (CI 20.6; 114.6; p = 0.0018)***	23.5 (CI-7.4; 64.7)
	Pz-O1	8.0 (CI -26.6; 58.9)	-10.1 (CI -38.2; 30.8)	**-42.0 (CI -60.2; -15.4; p = 0.0056)***	**-34.8 (CI -55.2; -5.0; p = 0.0266)***	**-36.3 (CI -56.3; -7.3; p = 0.0197)***

	Pz-O2	-16.2 (CI-46.5; 31.3)	-7.6 (CI -40.7; 43.9)	-24.5 (CI -51.7; 18.0)	-17.8 (CI-47.1; 27.7)	-34.5 (CI-57.9; 1.7)
**Eyes open Day 12 (multiple dose)**						
		**ENX-102 0.5 mg**	**ENX-102 1.0 mg**	**ENX-102 1.5 mg**	**ENX-102 2.0 mg**	**ENX-102 5.0 mg**
Delta-power (1.5 < 6.0 Hz)	Fz-Cz	3.1 (CI -28.8; 49.4)	**-36.4 (CI -57.0; -5.9; p = 0.0245)***	-24.8 (CI -47.7; 8.0)	-26.3 (CI -49.7; 8.0)	-15.3 (CI -40.8; 21.3)
	Pz-O1	-8.3 (CI -31.0; 21.8)	-24.3 (CI -44.5; 3.2)	**-31.2 (CI -48.2; -8.6; p = 0.0108)***	**-32.4 (CI -48.9; -10.4; p = 0.0072)***	**-41.8 (CI -56.1; -22.8; p = 0.0003)***
	Pz-O2	1.4 (CI -24.8; 36.8)	-23.8 (CI-44.9; 5.5)	**-38.2 (CI-54.3; -16.4l ; p = 0.0024)***	**-34.0 (CI -50.8; -11.5; p = 0.065)***	**-51.3 (CI -63.8; -34.5; p < 0.0001)***
Theta-power (6.0 < 8.5 Hz)	Fz-Cz	-19.6 (CI -41.7; 10.9)	**-56.7 (CI -69.2; -39.0; p < 0.0001)***	**-57.4 (CI -68.9; -41.6; p < 0.0001)***	**-54.9 (CI -67.3; -37.8; p < 0.0001)***	**-59.9 (CI -70.7; -45.1; p < 0.0001)***
	Pz-O1	-18.2 (CI-43.4; 18.3)	-28.9 (CI -52.4; 6.2)	**-44.7 (CI -61.7; -20.2; p = 0.0022)***	**-45.3 (CI -62.1; -21.0; p = 0.0018)***	**-61.2 (CI -73.1; -44.1; p < 0.0001)***
	Pz-O2	6.3 (CI -25.5; 51.8)	-24.3 (CI -48.5; 11.2)	**-47.9 (CI -63.4; -25.7; p = 0.0006)***	**-49.3 (CI -64.5; -27.7; p = 0.0004) ***	**-66.5 (CI -76.4; -52.3; p < 0.0001) ***
Alpha-power (8.5 < 12.5 Hz)	Fz-Cz	-15.1 (CI -44.0; 28.6)	-31.5 (CI -56.0; 6.6)	**-55.3 (CI -70.4; -32.5; p = 0.0003) ***	**-44.7 (CI -63.5; -16.4; p = 0.0061) ***	**-59.3 (CI -73.1; -38.4; p < 0.0001)***
	Pz-O1	-18.8 (CI -49.1; 29.4)	-25.9 (CI -55.5; 23.3)	**-47.1 (CI -67.1; -15.1; p = 0.0096)***	**-44.2 (CI -64.9; -11.1; p = 0.0154)***	**-54.6 (CI -71.6; -27.6; p = 0.0014)***
	Pz-O2	-14.5 (CI -44.0; 30.4)	-18.7 (CI -48.5; 28.5)	**-45.5 (CI -64.4; -16.4; p = 0.0064)***	**-46.0 (CI -64.6; -17.8; p = 0.0050)***	**-58.1 (CI -72.5; -36.2; p = 0.0001)***
Beta-power (12.5 < 30.0 Hz)	Fz-Cz	**37.4 (CI 1.6; 85.9; p = 0.0398)***	9.8 (CI -20.6; 51.9)	-2.7 (CI -27.7; 31.0)	**54.8 (CI 15.0; 108.4; p = 0.0049)***	**50.1 (CI 11.4; 102.3; p = 0.0087)***
	Pz-O1	-4.1 (CI -25.8; 23.9)	25.5 (CI -3.5; 63.3)	-15.0 (CI -33.7; 8.9)	6.5 (CI 16.5; 35.9)	8.5 (CI -15.0; 38.5)
	Pz-O2	2.5 (CI -24.8; 39.8)	22.2 (CI -11.8; 69.2)	-10.7 (CI -34.4; 21.5)	5.3 (CI -22.1; 42.5)	-9.5 (CI -33.1; 22.4)
Gamma-power (30.0 < 40.0 Hz)	Fz-Cz	23.0 (CI -11.0; 70.0)	-13.7 (CI -38.2; 20.5)	23.7 (CI-8.6; 67.4)	**46.6 (CI 8.3; 98.5; p = 0.0145)***	**85.1 (CI 36.7; 150.5; p = 0.0002)***
	Pz-O1	-2.4 (CI -33.9; 44.3)	29.1 (CI -11.1; 87.7)	-21.9 (CI -46.4; 13.8)	-19.1 (CI -44.4; 17.7)	-12.9 (CI -40.2; 26.9)
	Pz-O2	-11.2 (CI-46.8; 48.0)	-7.2 (CI-44.9; 56.4)	0.2 (CI -38.8; 64.0)	-9.7 (CI -44.6; 47.2)	-35.8 (CI -60.7; 4.8)
**Eyes closed Day 12 (multiple dose)**						
		**ENX-102 0.5 mg**	**ENX-102 1.0 mg**	**ENX-102 1.5 mg**	**ENX-102 2.0 mg**	**ENX-102 5.0 mg**
Delta-power (1.5 < 6.0 Hz)	Fz-Cz	**-29.5 (CI -48.9; -2.7; p = 0.0340)***	**-52.4 (CI -65.9; -33.4; p < 0.0001)***	**-40.8 (CI -57.2; -18.2; p = 0.0021)***	**-38.5 (CI -55.6; -14.8; p = 0.0044)***	**-45.6 (CI -60.7; -24.7; p = 0.0005)***
	Pz-O1	-26.3 (CI -45.7; 0.0)	**-42.8 (CI -58.6; -20.9; p = 0.0012)***	**-45.5 (CI -59.9; -26.1; p = 0.0002) ***	**-42.8 (CI -57.7; -22.6; p = 0.0005) ***	**-65.4 (CI -74.5; -53.0; p < 0.0001)***
	Pz-O2	**-26.7 (CI -46.1; -0.1; p = 0.0490)***	**-33.3 (CI -51.3; -8.6; p = 0.0128)***	**-38.6 (CI -54.8; -16.5; p = 0.0025)***	**-39.5 (CI -55.3; -17.9; p = 0.0017)***	**-65.8 (CI -74.8; -53.7; p < 0.0001)***
Theta-power (6.0 < 8.5 Hz)	Fz-Cz	-26.7 (CI -48.9; 5.1)	**-49.2 (CI -64.9; -26.6; p = 0.0006)***	**-60.6 (CI -72.5; -43.4; p < 0.0001)***	**-68.6 (CI -78.1; -54.9; p < 0.0001)***	**-72.9 (CI -81.1; -61.2; p < 0.0001)***
	Pz-O1	-13.9 (CI -40.2; 23.9)	-21.2 (CI -45.6; 14.3)	**-45.8 (CI -62.2; -22.2; p = 0.0014)***	**-58.3 (CI -71.0; -40.2; p < 0.0001)***	**-67.5 (CI -77.4; -53.3; p < 0.0001)***
	Pz-O2	13.2 (CI -21.5; 63.4)	-14.0 (CI -40.7; 24.7)	**-45.2 (CI -62.0; -21.0; p = 0.0019)***	**-59.8 (CI -72.2; -42.1; p < 0.0001)***	**-69.3 (CI -78.7; -55.7; p < 0.0001)***
Alpha-power (8.5 < 12.5 Hz)	Fz-Cz	-22.0 (CI -52.5; 28.0)	-27.0 (CI -56.0; 21.0)	**-56.4 (CI -73.5; -28.2; p = 0.0018)***	**-58.5 (CI -74.8; -31.9; p = 0.0009)***	**-58.2 (CI -74.6; -31.1; p = 0.0011)***

	Pz-O1	-21.7 (CI -47.3; 16.3)	-8.4 (CI -38.7; 36.8)	**-49.5 (CI -66.1; -24.8; p = 0.0013)***	**-46.1 (CI -63.8; -19.7; p = 0.0032)***	**-46.7 (CI -64.1; -20.8; p = 0.0026)***
	Pz-O2	-7.7 (CI -36.5; 34.1)	7.2 (CI -26.5; 56.3)	**-48.0 (CI -64.4; -24.2; p = 0.0011)***	**-53.4 (CI -68.0; -32.2; p = 0.0002)***	**-45.8 (CI -62.7; -21.3; p = 0.0019)***
Beta-power (12.5 < 30.0 Hz)	Fz-Cz	25.8 (CI -7.1; 70.4)	16.8 (CI -13.9; 58.5)	-7.9 (CI -31.8; 24.3)	29.8 (CI -3.9; 75.4)	21.4 (CI -10.1; 63.9)
	Pz-O1	-5.5 (CI -25.9; 20.5)	25.9 (CI -1.1; 60.3)	-12.3 (CI -30.9; 11.4)	5.9 (CI -16.6; 34.4)	12.1 (CI -11.7; 42.2)
	Pz-O2	1.6 (CI -21.0; 30.7)	24.1 (CI -3.7; 59.8)	-5.8 (CI -26.6; 21.1)	3.6 (CI -19.3; 33.1)	0.5 (CI -21.7; 29.0)
Gamma-power (30.0 < 40.0 Hz)	Fz-Cz	20.7 (CI -10.8; 63.2)	-17.0 (CI -38.3; 11.6)	12.3 (CI -15.8; 49.8)	**36.1 (CI 2.0; 81.7; p = 0.0366)***	**67.1 (CI 25.3; 122.8; p = 0.0008)***
	Pz-O1	-0.7 (CI -31.6; 44.2)	30.3 (CI -10.2; 88.9)	-24.1 (CI -46.7; 8.1)	-12.9 (CI -38.7; 23.6)	-7.0 (CI -34.6; 32.1)
	Pz-O2	-21.6 (CI-50.0; 22.9)	-19.4 (CI -48.2; 25.5)	-8.8 (CI -41.7; 42.6)	-21.7 (CI -49.7; 21.8)	**-37.3 (CI-59.6; -2.6; p = 0.0384)***

CI: Confidence Interval: Fz-Cz: Frontal-central electrode position in EEG; Hz: Hertz, mg: Milligram; Pz-O1: Parietal-occipital electrode position 1 in EEG; PzO2: Parietal-occipital electrode position 2 in EEG.

Table S7: qEEG results of the human multiple ascending dose study eyes open and eyes closed states at Day 1 and at Day 12. Statisticallty=significant changes ($p < 0.05$),baseline corrected and compared with placebo, are indicated in bold with an asterisk (*). Confidence intervals represent 95% CI.
